# The *CsMYB36-CsSWEET17* module mediates the calcium-induced sucrose accumulation in citrus

**DOI:** 10.1093/hr/uhaf175

**Published:** 2025-07-16

**Authors:** Xiawei Sheng, Mengdi Li, Yanrou Luo, Zuolin Mao, Xiawan Zhai, Ji-Hong Liu, Chunlong Li

**Affiliations:** National Key Laboratory for Germplasm Innovation & Utilization of Horticultural Crops, College of Horticulture and Forestry Sciences, Huazhong Agricultural University, Wuhan 430070, China; National Key Laboratory for Germplasm Innovation & Utilization of Horticultural Crops, College of Horticulture and Forestry Sciences, Huazhong Agricultural University, Wuhan 430070, China; National Key Laboratory for Germplasm Innovation & Utilization of Horticultural Crops, College of Horticulture and Forestry Sciences, Huazhong Agricultural University, Wuhan 430070, China; National Key Laboratory for Germplasm Innovation & Utilization of Horticultural Crops, College of Horticulture and Forestry Sciences, Huazhong Agricultural University, Wuhan 430070, China; National Key Laboratory for Germplasm Innovation & Utilization of Horticultural Crops, College of Horticulture and Forestry Sciences, Huazhong Agricultural University, Wuhan 430070, China; National Key Laboratory for Germplasm Innovation & Utilization of Horticultural Crops, College of Horticulture and Forestry Sciences, Huazhong Agricultural University, Wuhan 430070, China; Hubei Hongshan Laboratory, Wuhan 430070, China; National Key Laboratory for Germplasm Innovation & Utilization of Horticultural Crops, College of Horticulture and Forestry Sciences, Huazhong Agricultural University, Wuhan 430070, China; Hubei Hongshan Laboratory, Wuhan 430070, China

## Abstract

Sugar content serves as a crucial determinant of fruit flavor quality and nutritional value. Calcium plays extensive regulatory roles in fruit development and quality formation, yet the molecular mechanisms underlying calcium-mediated sugar accumulation remain poorly understood. In this study, we demonstrate that calcium treatment enhances sugar accumulation in both citrus fruits and calli, concomitant with upregulated expression of the sucrose transporter gene *CsSWEET17*. Functional characterization revealed that the membrane-localized CsSWEET17 protein exhibits sucrose transport activity. Transgenic overexpression of *CsSWEET17* in citrus juice sacs, calli and heterologous tomato systems consistently elevated sucrose levels. Conversely, suppression of *CsSWEET17* expression through either virus-induced gene silencing or RNA interference significantly reduced sucrose content in citrus. Further investigation identified CsMYB36 as a calcium-responsive transcription factor that directly activates *CsSWEET17* expression. Transgenic validation demonstrated that both calcium signaling and CsMYB36-mediated sucrose accumulation strictly depend on *CsSWEET17* transcriptional regulation. Our findings elucidate a novel calcium-MYB36-SWEET17 regulatory module controlling sucrose accumulation, providing molecular insights into calcium-based strategies in citrus quality improvement and informing fundamental mechanisms of sugar transporter regulation in fruit crops.

## Introduction

Citrus stands as the world's most extensively cultivated fruit crop, renowned for its substantial economic significance and nutritional value in global horticulture [1–3]. Fruit quality, particularly the sugar–acid equilibrium, serves as a critical parameter influencing citrus market competitiveness and consumer preference [4]. Among the quality determinants, sucrose accumulation stands out as the predominant metabolite marker of internal quality, comprising over 66% of total soluble sugars in mature citrus fruits, and occurring at twice the rate observed for glucose or fructose accumulation [5]. This disaccharide undergoes phloem-mediated transport from photosynthetic source tissues to sink organs, making the molecular regulation of sucrose translocation and compartmentalization as the key research priority for citrus quality improvement.

As the principal transport metabolite, sucrose requires specialized transmembrane transport mechanisms during phloem unloading and subsequent cellular compartmentalization processes mediated by dedicated sucrose transporter (SUT) proteins [6, 7]. Thus, transporter proteins responsible for the transmembrane transport of soluble sugars are essential for sugar movement between sources and sink tissues. To date, three sugar transporter protein families have been identified in plants: monosaccharide transporters (MSTs), SUTs and sugars will eventually be exported transporters (SWEETs) [8]. Among that, SWEETs are a novel class of sugar transporters that can transport sugars bidirectionally and facilitate sugar diffusion along concentration gradients [9], with different subfamilies having selective preferences for monosaccharides or disaccharides. For instance, the cell membrane-localized SlSWEET7a and SlSWEET14 have been shown to regulate sugar transport and storage in tomato fruit [10]. The pineapple sugar transporter AcSWEET10 functions in glucose transport [11]. OsSWEET11, OsSWEET15 and the aleurone-localized OsSUTs provide sucrose to aleurone [12]. In maize, ZmSWEET11/13b exports sucrose to the apoplast, while ZmSTP3, ZmSWEET3a/4c (MSTs), ZmSUT1 and ZmSWEET11/13a (SUTs) participate in sucrose or hexose recovery in the apoplast after extracellular enzymatic hydrolysis in basal endosperm transfer cells [13]. AtSWEET17 has been shown to localize in the vacuole of roots, where it plays a key role in the bidirectional transport of fructose across the root cytoplasm, responding to stress or drought by maintaining cytoplasmic fructose homeostasis [14–16]. Despite these advances, the functional characterization of SWEET proteins in citrus remains a crucial knowledge gap, particularly regarding their subcellular targeting diversity, substrate selectivity patterns and integration with fruit sugar metabolism networks.

Transcription factors (TFs) serve as master regulators of gene expression, governing fundamental biological processes including developmental patterning, quality trait establishment and signal transduction cascades [17–21]. Among these, MYB transcription factors constitute one of the largest and most versatile plant-specific TF families, characterized by their evolutionarily conserved MYB DNA-binding domain. These regulators orchestrate diverse physiological programs ranging from reproductive development and cell cycle modulation to stress adaptation and specialized metabolite biosynthesis. MYB36, a functionally prominent member, has been extensively characterized for its pleiotropic regulatory capacities. In *Arabidopsis* roots, MYB36 coordinates a transcriptional network directing lignin and suberin biosynthesis [22]. Similarly, OsMYB36 in rice transcriptionally regulates over 1000 genes implicated in casparian strip formation and suberin deposition [23]. In *Salvia miltiorrhiza*, MYB36 promotes the accumulation of tanshinones while reducing phenolic acid levels [24]. An MYB transcription factor StAN1 in potato can directly binds to the promoters of sucrose hydrolysis genes *SUSY1* and *INV2* to positively regulate their expression and sucrose content [25]. In oriental melon, CmMYB44 represses the transcription of two key genes, *sucrose-phosphate synthase 1* (*CmSPS1*) and *ACC oxidase 1* (*CmACO1*), involved in sucrose and ethylene accumulation respectively [26]. Apple MdMYB305 and MdMYB10 further exemplify MYB functional complexity, modulating metabolic partitioning between soluble sugars and anthocyanins through competitive promoter binding with MdbHLH33 [27]. Regulatory interactions between transcription factors and SWEET transporters have also been documented. For instance, MYB33 enhances the expression of *SWEET11* and *SWEET21* to regulate the sucrose transport from cotyledons to hypocotyls [9]. In grape, a well-defined regulatory hierarchy governs hexose homeostasis. Specifically, during the pre-veraison phase, VvERF105 suppresses the expression of *VvSWEET15*, thereby limiting hexose accumulation. Conversely, VvNAC72 activates *VvSWEET15* to facilitate accelerated sugar import in post-veraison stage [28]. Notably, during root-knot nematode infection, SlDOF9 has been shown to modulate the expression of *SlSWEET17* in tomato plants [29]. However, the mechanistic involvement of MYB transcription factors in citrus sucrose accumulation remains unresolved, representing a limitation in understanding transcriptional regulation of citrus sweetness quality.

Calcium (Ca^2+^) is not only a structural component in organisms, but more importantly, it is a widely recognized intracellular second messenger, central to regulating nearly all cellular and plant responses to environmental and internal changes [30–32]. As one of the essential mineral elements in plants, optimal Ca^2+^ supplementation significantly enhances fruit quality parameters including firmness, shelf-life and sugar metabolism [33–36]. Ca^2+^ antagonists have been shown to inhibit sugar uptake in *Arabidopsis* seedlings [37]. Calmodulin proteins, as intracellular Ca^2+^ receptors, mediate the effects of Ca^2+^ on carbohydrate metabolism, secretion and movement. In grapevines deficient in Ca^2+^, the activities of rubisco, sucrose–phosphate synthase, sucrose synthase (SS) and fructose-1,6-bisphosphatase are elevated, while the activities of ADP-glucose pyrophosphorylase and ribulose-1,5-bisphosphate carboxylase are reduced [38]. These findings collectively establish a direct link between Ca^2+^ signaling and sucrose accumulation [39]. However, the downstream Ca^2+^ signaling mechanisms governing fruit quality in citrus, particularly transcriptional regulation of sugar transporters and metabolic enzymes, are remain largely unexplored.

This study demonstrates that Ca^2+^ treatment significantly enhances sucrose accumulation in both citrus fruits and calli. Comparative transcriptome profiling of Ca^2+^-treated tissues identified the plasma membrane-localized SUT CsSWEET17 as a key mediator of Ca^2+^-induced sucrose enrichment. Yeast one-hybrid (Y1H) screening further revealed that the transcription factor CsMYB36 directly binds to the CsSWEET17 promoter, activating its transcriptional activity and driving sucrose accumulation in transgenic citrus systems. Our findings uncover a previously uncharacterized Ca^2+^-responsive regulatory mechanism involving the CsMYB36–CsSWEET17 axis, providing both fundamental insights into sucrose transport regulation and practical strategies for fruit quality enhancement through Ca^2+^-based interventions.

## Results

### The Ca^2+^-induced accumulation of sucrose is associated with the high expression of *CsSWEET17*

**Figure 1:**
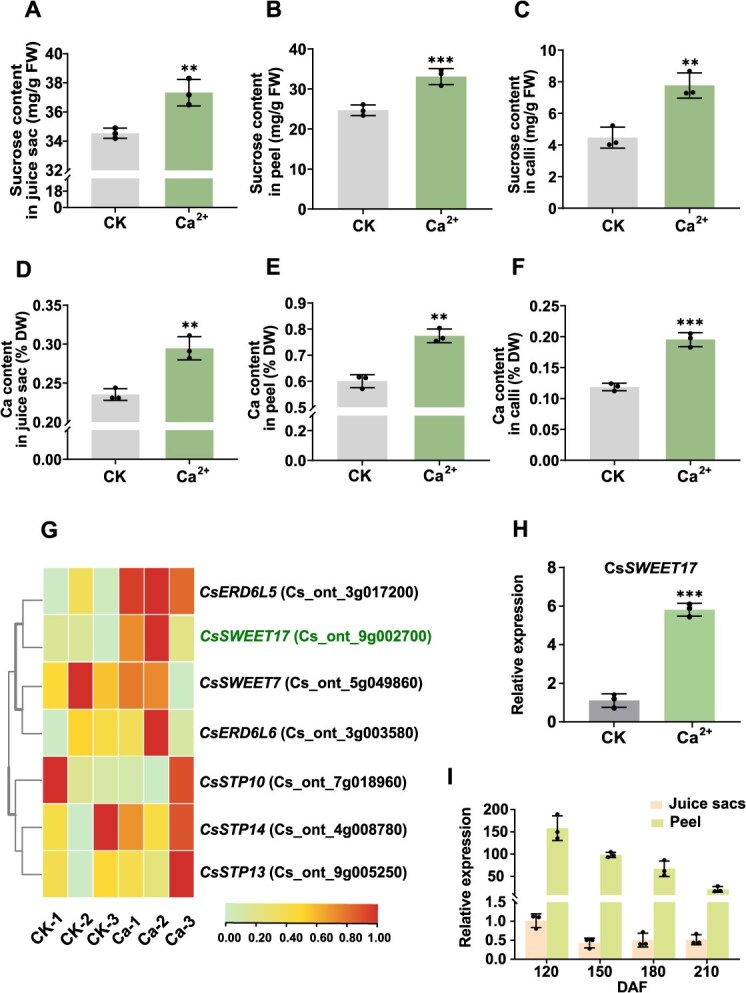
Calcium induces sucrose accumulation in citrus and the discovery of *CsSWEET17.* (A-B) Calcium induces the higher sucrose content in juice sacs (A), peel (B) and citrus calli (C). (D-F) The calcium content assay in control and calcium-treated citrus juice sacs (D), peel (E) and calli (F). (G) Heatmap displaying the expression patterns of DEGs in calcium-treated (Ca-1-3) and control (CK-1-3) calli. The values represent log-transformed FPKM (Fragments Per Kilobase Million) levels. (H) RT-qPCR confirmed the transcript levels of *CsSWEET17* in calcium-treated calli. (I) Expression pattern of *CsSWEET17* in juice sacs and peel during citrus fruit development. Error bars represent the standard error (SE) based on three biological replicates. Asterisks indicate statistically significant differences compared with the control group (Student's t-test: ^**^P < 0.01, ^***^P < 0.001). CK, control; FW, fresh weight; DW, dry weight; DAF, days after flowering.

To investigate the function of Ca^2+^ on fruit quality formation, we treated Navel orange (*Citrus sinensis* Osb. var. *brasliliensis* Tanaka) with Ca^2+^ during the fruit development stages and measured the sugar content in the fruit. The result indicated a significant increase in fructose, glucose and sucrose contents across both juice sacs and peels of citrus fruits (Fig. 1A and B, Supplementary Fig. S1A–D). To further elucidate the underlying mechanisms of Ca^2+^-mediated sugar accumulation, we treated calli tissue from the ‘Guoqing No. 1’ variety with Ca(NO_3_)_2_. After 15 days, the sugar content analysis revealed that fructose, glucose and sucrose content in Ca-treated calli was significantly higher than that in the control sample (Fig. 1C and Supplementary Fig. S1E and F). Notably, sucrose accumulation demonstrated the most substantial enhancement across these tissues, exhibiting a statistically significant elevation compared to glucose and fructose levels. We also measured the Ca^2+^ content in Ca^2+^-treated juice sacs, peel and calli, all of which exhibited a significant increase compared to untreated controls (Fig. 1D–F), demonstrating that Ca-induce sucrose accumulation is conserved in citrus fruit and calli. To uncover the potential molecular regulatory mechanism by which Ca-treatment improves fruit sugar content, we performed RNA-sequencing (RNA-seq) analysis of Ca-treated and control citrus calli samples with three biological replicates. Through differential expression gene (DEG) analysis, we identified candidate transporter genes potentially involved in regulation of sugar content. Among these, most sugar-related transporters showed upregulation pattern (Fig. 1G), which were selected as candidate genes. The RT-qPCR analysis of seven genes revealed that the expression of *CsSWEET17* was most notably upregulated in Ca-treated samples compared to the control (Fig. 1H, Supplementary Fig. S2A–F).

**Figure 2:**
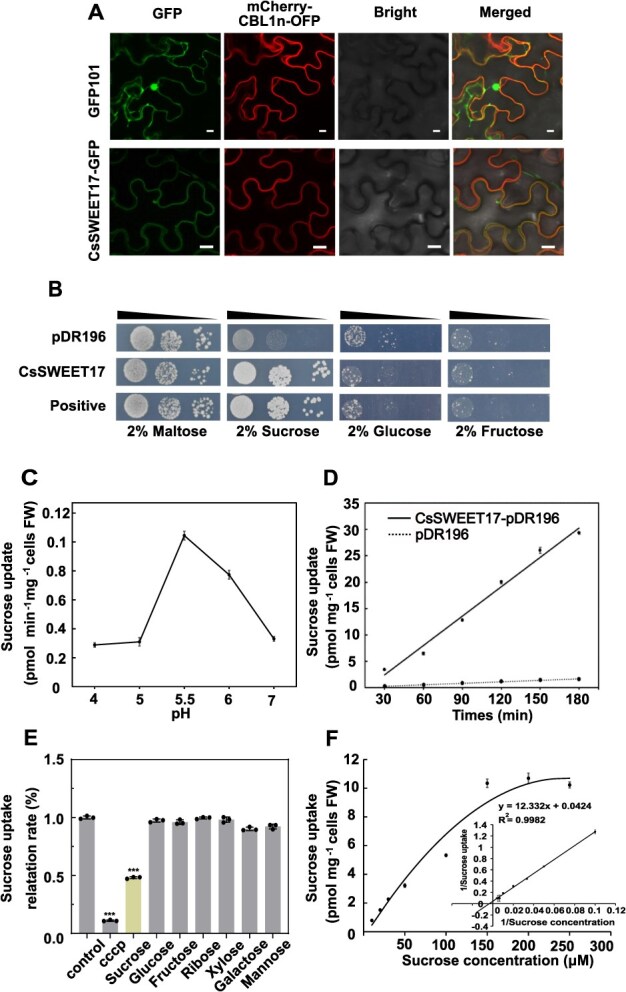
Subcellular localization of CsSWEET17 and verification of its sucrose transport activity. (A) Subcellular localization of CsSWEET17 transiently expressed in *Nicotiana benthamiana* leaves, co-localizing with a plasma membrane marker protein (mCherry-CBL1n-OFP). Scale bars = 10 μm. (B) CsSWEET17 complements sucrose and hexose transporter-deficient yeast strain CSY4000, as evidenced by growth on selective media compared to the negative controls, pDR196 empty vector; positive controls, StSUT1-pDR196. The yeast grew for 3 days before being photographed. (C) Investigation of pH-dependent sucrose uptake by CsSWEET17 in yeast system, measured by ^14^C-Suc incorporation. (D) Time-dependent sucrose uptake by CsSWEET17, with pDR196 (empty vector) as a negative control. (E) In the absence of competing sugars and metabolic inhibitors, ^14^C-Suc uptake by CsSWEET17 was measured, with control conditions set at 100%. (F) Michaelis–Menten kinetics analysis of CsSWEET17 revealed a *Km* of 290.84 μM and a *Vmax* of 23.584 pmol Sucrose min^−1^ mg^−1^ cells FM (fresh weight). Data analysis was conducted using R software (https://rpubs.com/RomanL/6752). Values represent means ± SE (*n* = 3). Significant differences were determined using one-way ANOVA with Tukey’s test (^***^P < 0.001).

Based on protein sequence alignment of the *SWEET17* with several plant species *(Arabidopsis thaliana*, *Glycine max* and *Zea mays*), CsSWEET17 is highly conserved in the plants and harbors seven typical transmembrane domains (TMs) (Supplementary Fig. S2G and H). We further investigated the spatial and temporal expression patterns of *CsSWEET17* in the citrus juice sacs and peel using RT-qPCR. The results demonstrated that the expression of *CsSWEET17* in the peel was consistently much higher than that in the juice sacs, and its expression was slightly declined over time in both tissues (Fig. 1I). Considering that most vascular bundles are clustered in the peel [40], this implies that CsSWEET17 may be involved in sugar unloading from the peel to the juice sacs.

### CsSWEET17 is localized to the plasma/vacuolar membrane and has sucrose transport activity

To determine the subcellular localization of CsSWEET17, the *CsSWEET17*-GFP fusion construct was transiently expressed in tobacco epidermal cells using *Agrobacterium tumefaciens*. The pRI101-GFP (empty vector, EV) served as a control. As shown in Fig. 2A, CsSWEET17-Green Fluorescent Protein (GFP) signal was observed at the plasma membrane, co-localizing with the mCherry-CBL1n-OFP plasma membrane marker. These findings provide evidence that CsSWEET17 is localized to the plasma membrane. Previous studies have reported that the SWEET17 protein is also located to the vacuolar membrane [14, 16]. To validate this observation, we conducted subcellular localization analysis of protoplasts and released vacuoles. Notably, CsSWEET17 displayed a polytopic localization pattern, being distributed across the plasma membrane, tonoplast and small vesicles (Supplementary Fig. S3A). This multi-compartmental distribution mirrors the behavior of tomato SlSWEET17 as recently reported [29], indicating a conserved mechanism of membrane trafficking among SWEET17 homologs between citrus and tomato.

The hexose and sucrose transport-deficient yeast strain CSY4000 was further applied to detect the transport substrate of CsSWEET17. The yeast expression vector pDR196 was used to express *CsSWEET17*, with the *StSUT1* and empty pDR196 vector serving as the positive and negative control respectively. As expected, all strains grew similarly on 2% (w/v) maltose medium. However, compared to the EV control cells, the yeast with the CsSWEET17 and StSUT1 can grow well on 2% (w/v) sucrose medium, but show no growth difference on either 2% (w/v) fructose or glucose sugar medium (Fig. 2B). This result demonstrates that CsSWEET17 functions as an SUT. Given the multi-subcellular localization patterns of CsSWEET17, we also employed a tonoplast membrane yeast system and esculin to simulate CsSWEET17's sucrose transport activity based on previous report [41]. As shown in Supplementary Fig. S3B, the esculin signal was presented in the vacuole of yeast cells with the expression of *CsSWEET17* and positive control *CsTST2*, implying the sucrose transport activity of CsSWEET17 in both plasma and vacuolar membrane.

To further analyze the transport properties of CsSWEET17, ^14^C-labeled sucrose was used in yeast uptake assay. As shown in Fig. 2C and D, under optimal pH 5.5 condition, sucrose uptake rate by CSY4000 yeast cells expressing CsSWEET17 was significantly higher than that of control. The competitive uptake of ^14^C-sucrose in the presence of a 100-fold excess of unlabeled sugars was evaluated to assess the substrate specificity of CsSWEET17. The results showed that only non-radioactive sucrose reduced the uptake of ^14^C-sucrose to 52% of the control, while other substrates, including glucose, fructose, ribose, xylose, galactose and mannose, had no effect. This indicates that *CsSWEET17* is a sucrose-specific transporter (Fig. 2E). Furthermore, low concentrations of the proton uncoupler carbonyl cyanide m-chlorophenyl hydrazone significantly reduced ^14^C-sucrose uptake to 10% of control uptake (Fig. 2E), suggesting that sugar uptake via CsSWEET17 is proton-coupled process. The consistent ^14^C-sucrose uptake results were also observed in the *Xenopus laevis* oocytes with the expression of CsSWEET17 (Supplementary Fig. S3C and D). The Michaelis–Menten kinetics of CsSWEET17 was analyzed by measuring ^14^C-sucrose uptake across different sucrose concentrations. Nonlinear regression analysis revealed a *Km* value of approximately 290.84 μM and a maximum uptake rate (*Vmax*) of 23.584 pmol sucrose min^−1^ mg^−1^ cells fresh weight at pH 5.5 (Fig. 2F). Collectively, these findings indicate that membrane-localized CsSWEET17 specifically transports sucrose.

### CsSWEET17 enhances the sucrose content in citrus and tomato fruit

**Figure 3:**
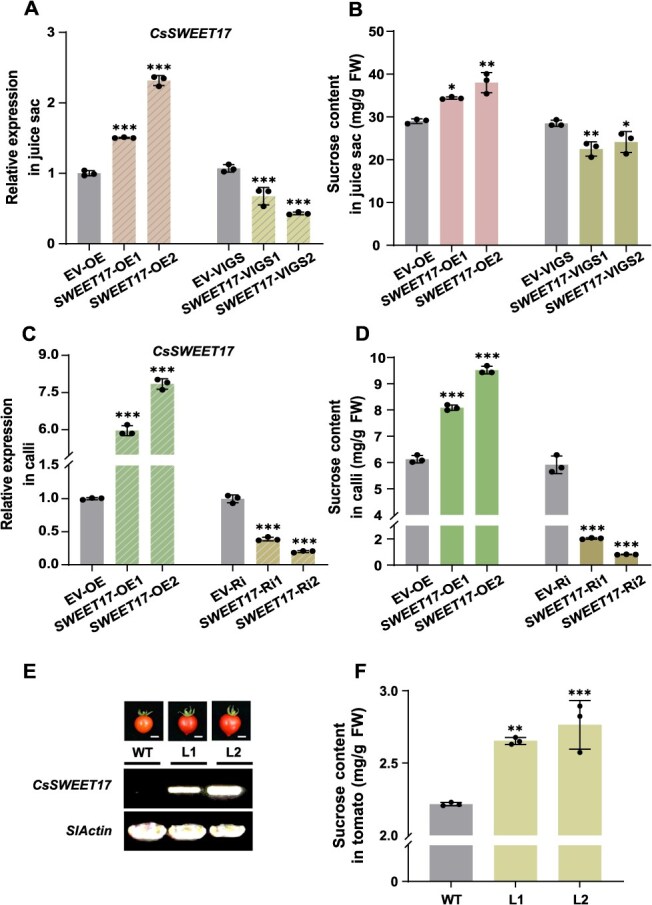
CsSWEET17 is contributed to the accumulation of sucrose. (A-B) The transcript expression level of *CsSWEET17* (A) and sucrose content (B) in the transgenic juice sacs. Overexpression empty vector (EV-OE) and TRV2 empty vector (EV-VIGS) refer to the control. (C-D) The transcript expression level of *CsSWEET17* (C) and sucrose content (D) in the transgenic citrus calli. Overexpression empty vector (EV-OE) and RNAi empty vector (EV-Ri) refer to the control. (E) The fruit photos and analysis of *CsSWEET17* expression in overexpression transgenic tomato fruit lines (L1 and L2). *SlActin* works as internal reference. Scale bars = 1 cm. (F) Sucrose content in WT and overexpression transgenic tomato fruits. All the above results, each point represents an independent biological repeat and error bars means ± SE (*n* = 3). FW, Fresh weight; WT, wild type. Asterisks indicate statistically significant differences compared with controls (one-way ANOVA with Tukey’s test, ^*^P < 0.05, ^**^P < 0.01, ^***^P < 0.001).

To further elucidate the function of CsSWEET17 in sucrose accumulation *in planta*, recombinant constructs C*sSWEET17*-pK7WG2D and *CsSWEET17*-pTRV2 were infiltrated into citrus juice sacs to achieve overexpression (OE) and virus-induced gene silencing (VIGS) of *CsSWEET17*, respectively. The target gene expression level and infiltration success were confirmed by RT-qPCR (Fig. 3A). The results showed that sucrose concentrations in *CsSWEET17*-OE samples were significantly higher than that in the control group, whereas *CsSWEET17*-VIGS juice sacs exhibited the opposite phenotype (Fig. 3B). Subsequently, recombinant constructs *CsSWEET17*-pK7WG2D (OE) and *CsSWEET17*-pK7GWIWG2D (RNAi) were used for Agrobacterium-mediated transformation of citrus calli. RT-qPCR analysis identified two OE lines (*SWEET17*-OE1 and *SWEET17*-OE2) and two RNA interference (RNAi) lines (*SWEET17*-Ri1 and *SWEET17*-Ri2), as well as calli transformed with the EV serving as control (Fig. 3C). The OE lines exhibited approximately 1.5 times increase in sucrose content compared to the control group (Fig. 3D). Conversely, the RNAi lines displayed a reduction in sucrose accumulation, showing the opposite phenotype (Fig. 3D). Additionally, the recombinant construct *CsSWEET17*-pK7WG2D was transformed into tomato cotyledons to generate heterologous OE transgenic materials. RT-PCR analysis yielded homozygous transgenic T2 lines (Fig. 3E). Sugar content measurements of transgenic tomato fruits at the red ripening stage revealed that *CsSWEET17* significantly enhanced sucrose accumulation (Fig. 3F). These phenotypic results from *CsSWEET17*-transgenic citrus juice sacs, calli and tomato collectively demonstrate that CsSWEET17 serves as a positive regulator of sucrose accumulation in citrus fruit.

### Identification and expression pattern analysis of *CsMYB36*

**Figure 4:**
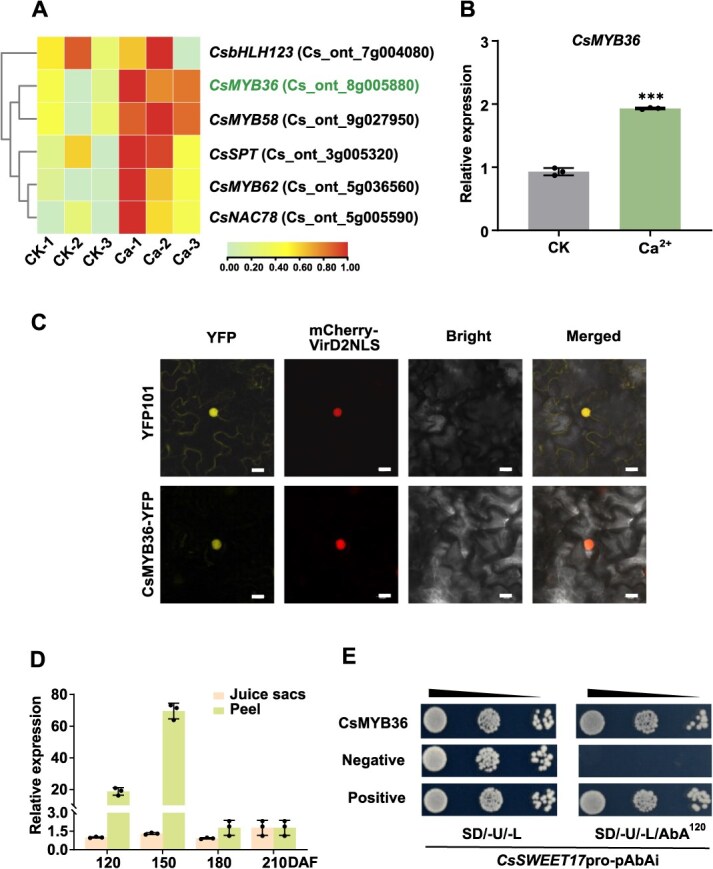
Screening and localization assay of CsMYB36. (A) Heatmap showing the expression patterns of significantly different transcription factors in calcium-treated and control (untreated) calli. The value was log of FPKM. (B) RT-qPCR analysis revealed significant upregulation of *CsMYB36* in calcium-treated calli compared to the control. This indicates a strong association between calcium treatment and the expression of CsMYB36. (C) Subcellular localization of CsMYB36 in *Nicotiana benthamiana* leaves was confirmed using transient expression and fluorescence microscopy. Co-localization with a nuclear marker (mCherry) demonstrated that CsMYB36 is localized in the nucleus. Scale bars = 10 μm. (D) Expression patterns of *CsMYB36* were analyzed during different stages of citrus fruit development in juice sacs and peel. (E) Y1H assay of CsMYB36 binding to CsSWEET17 promoter. At a concentration of 120 mg/L AbA, the self-activation of *CsSWEET17*pro-pAbAi was inhibited. The assay was performed on SD medium lacking Ura and Leu to select for interactions. Positive control: Interaction between pGBKT7-P53 and pGADT7-53. Negative control: CsSWEET17pro-pAbAi with pGADT7 empty vector. Significant interactions were observed at various AbA concentrations. Each experiment's results are represented as means ± SE (*n* = 3). Asterisks indicate statistically significant differences compared with controls (one-way ANOVA with Tukey’s test, ^***^P < 0.001). Days After Flowering (DAF) indicate the developmental stages of citrus fruit.

To investigate the molecular mechanism by which *CsSWEET17* promotes sucrose accumulation, we analyzed the promoter sequence within 1100 bp upstream of the *CsSWEET17* gene’s start codon by TBtools [42] (Supplementary Fig. S4A). Furthermore, six transcription factors (TFs) induced by Ca^2+^ treatment were identified from transcriptomic data (Fig. 4A). Among these, *CsMYB36* showed the most significant Ca-induced expression as confirmed by RT-qPCR method (Fig. 4B, Supplementary Fig. S4B–F). Multiple sequence alignment revealed that *CsMYB36* contains the conserved R2R3 MYB transcription factor domain as previous reported (Supplementary Fig. S4G) [43]. Subcellular localization assay with the nuclear marker protein further confirmed the nucleus-localization of CsMYB36 (Fig. 4C). Additionally, expression analysis showed that *CsMYB36* was highly expressed in the fruit peel, with its expression level decreasing during fruit development, which was similar to that of CsSWEET17 (Fig. 4D). To verify the binding ability of candidate TFs to *CsSWEET17* promoter, Y1H colonies’ growth was observed for two days on SD/-U-L medium supplemented with Aureobasidin A (AbA). The results indicated that only *CsMYB36* bound to the *CsSWEET17* promoter, achieving steady-state growth of yeast (Fig. 4E, Supplementary Fig. S4H). These findings support CsMYB36 as a potential upstream transcriptional regulator of *CsSWEET17*, warranting further investigation.

### CsMYB36 binds to the promoter of *CsSWEET17* and activates its expression

**Figure 5:**
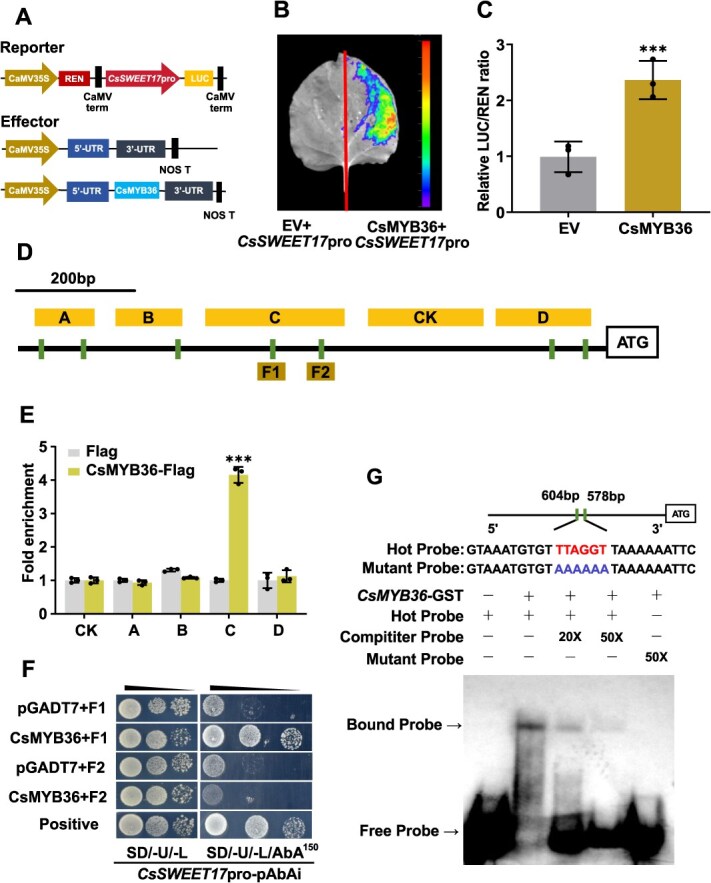
CsMYB36 binds to the *CsSWEET17*’s promoter. (A) Schematic diagram of the reporter and effector structure in the dual-LUC assay. (B) Dual-LUC reporter assay demonstrating the interaction between the *CsSWEET17* promoter and CsMYB36 in *Nicotiana benthamiana* leaves. (C) LUC assay results indicate that CsMYB36 enhances the transcriptional activity of the *CsSWEET17* promoter. The LUC/REN ratio, normalized to the empty vector control (set as 1), shows significant activation by CsMYB36. Error bars represent the standard error (SE) based on three biological replicates. Significant differences compared to controls are marked with ^***^P < 0.001 (Student's *t*-test). (D) Structural diagram of the CsMYB36 transcription factor binding site within the *CsSWEET17* promoter. Regions A-D represent segments for MYB36 binding assay via ChIP-qPCR (E, Supplementary Fig. S5) and Y1H experiment (F). F1 and F2 denote specific promoter regions tested in the Y1H experiment. (E) ChIP-qPCR analysis confirms the enrichment of *CsSWEET17* promoter sequence at the MYB36-FLAG protein, indicating direct binding and regulation. (F) Segmented Y1H experiments reveal specific interactions between CsMYB36 and promoter regions F1 and F2 of *CsSWEET17*. Positive controls (pGBKT7-P53 and pGADT7-53) validate the interaction detection method. (G) Electrophoretic Mobility Shift Assay (EMSA) demonstrates the binding of CsMYB36 to the *CsSWEET17* promoter. The upper sequence denotes the MYB36 binding element, while lower sequence indicates the mutant element. The binding site is located between 578 bp and 604 bp upstream of the promoter. Competition (20×, 50×) and mutation probes are shown, with + and − indicating presence or absence. The arrows indicate protein-DNA complexes (shift bands).

Given the stable yeast growth phenotype observed in the Y1H assay, a dual-luciferase (LUC) assay was conducted to elucidate how CsMYB36 regulates *CsSWEET17* expression (Fig. 5A). Compared to the control group (EV-62SK), tobacco leaves co-infiltrated with *CsMYB36*-62SK and *CsSWEET17*-0800pro exhibited significantly higher LUC activity (Fig. 5B). This result was consistent with LUC/REN enzyme activity measurements and in vivo leaf imaging (Fig. 5C), indicating that CsMYB36 positively regulates *CsSWEET17* expression by binding to its promoter. To identify the specific binding site of CsMYB36, the *CsSWEET17* promoter was divided into four fragments (labeled A, B, C and D, shown as rectangles in Fig. 5D) based on the locations of MRE, MYB and MBS binding motifs (Supplementary Fig. S4A). Each fragment, containing at least one of MYB-binding motif, was inserted into the pAbAi vector and co-transformed with *CsMYB36*-pGADT7 into Y1H yeast cell. Growth on SD/-U/-L plates containing 150 mg/L AbA revealed that CsMYB36 specifically bound to the *CsSWEET17*pro-C fragment (Supplementary Fig. S5). To confirm *in vivo* binding of *CsMYB36* to the *CsSWEET17* promoter, chromatin immunoprecipitation (ChIP)-qPCR was performed using transgenic citrus calli stably expressing 35S::CsMYB36-flag and a flag-EV as the control. A negative control region (CK) without binding motif was also included. ChIP-qPCR results showed significant enrichment of the C fragment in the immunoprecipitated samples from 35S::CsMYB36-flag calli (Fig. 5E). This fragment, located 578 bp upstream of the ATG start codon, contains an MRE and an MYBcore motif, which were designed as F1 and F2 for further confirmation by Y1H assay (Fig. 5D). The yeast growth result showed that CsMYB36 specifically bound to the F1 sequence of *CsSWEET17* promoter (Fig. 5F). In addition, an electrophoretic mobility shift assay (EMSA) confirmed the *in vitro* DNA-binding site of CsMYB36. Probes corresponding to the F1 region of the *CsSWEET17* promoter were designed, including labeled, unlabeled competitive and mutated probes. The probe movement confirmed specific binding of CsMYB36 to the region located 578–604 bp upstream of the *CsSWEET17* initiation codon (ATG). And this binding activity decreased with the addition of unlabeled competitive probes (Fig. 5G). In summary, these results demonstrate that *Cs*MYB36 directly binds to the *CsSWEET17* promoter, consistent with its role as a positive regulator of *CsSWEET17* transcription.

**Figure 6:**
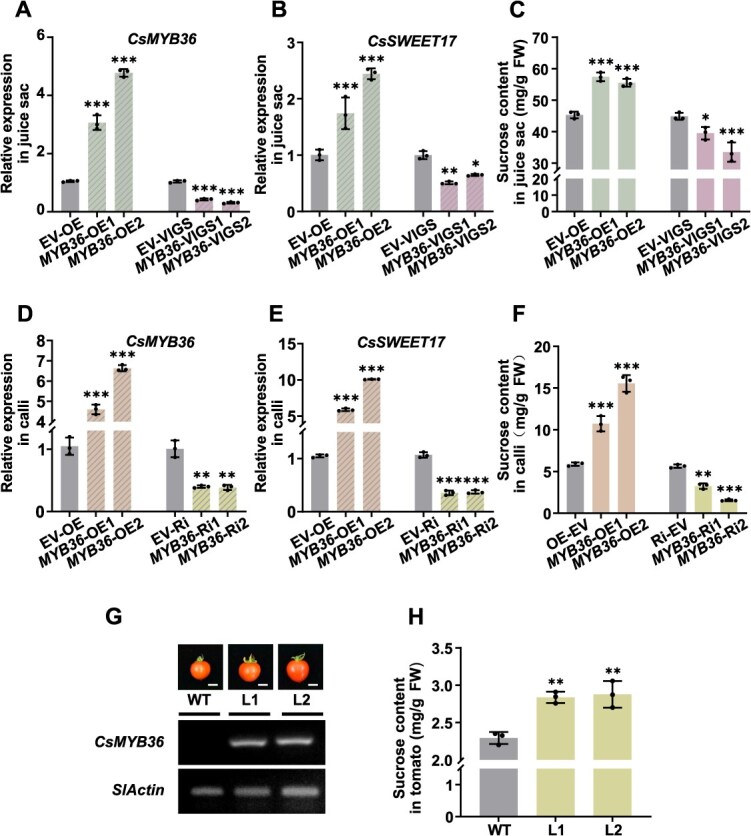
*CsMYB36* is associated with sucrose accumulation in both citrus and tomato. (A-C) The transcript expression level of *CsMYB36* (A) and *CsSWEET17* (B), and sucrose content (C) in the transgenic juice sacs. Overexpression empty vector (EV-OE) and TRV2 empty vector (EV-VIGS) refer to the control. (D-F) The transcript expression level of *CsMYB36* (D) and *CsSWEET17* (E), and sucrose content (F) in the transgenic citrus calli. Overexpression empty vector (EV-OE) and RNAi empty vector (EV-Ri) refer to the control. (G) The fruit photos and transcript analysis of *CsMYB36* expression in overexpression transgenic tomato fruits. Scale bars = 1 cm. *SlActin* works as internal reference. (H) Sucrose content in overexpression transgenic tomato fruits. L-1 and L-2, *CsMYB36*-overexpressing tomato lines; WT, Wild Type, refers to the control. All the above results, each point represents an independent biological repeat and error bars means ± SE (*n* = 3). Asterisks indicate statistically significant differences compared with controls (Student’s *t*-test, ^*^P < 0.05, ^**^P < 0.01, ^***^P < 0.001). FW, Fresh weight.

**Figure 7:**
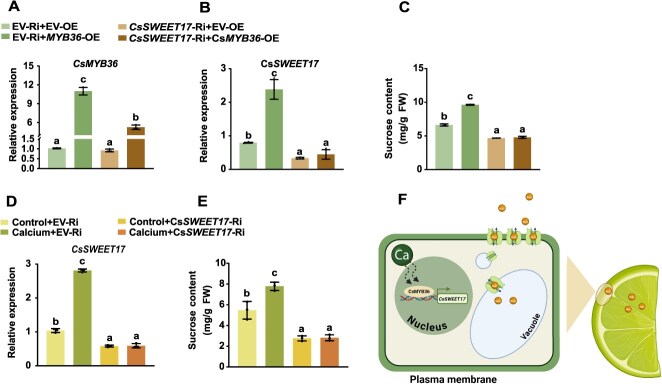
CsSWEET17 is essential for CsMYB36 and calcium induced sucrose accumulation in citrus. (A-C) Overexpression of *CsMYB36* was achieved in transgenic tissue-cultured citrus calli lines, including an empty vector control and a *CsSWEET17*-silenced background. The expression levels of *CsMYB36* (A) and *CsSWEET17* (B) were analyzed using RT-qPCR, and sucrose content was measured (C). (D-E) Calcium treatment was applied to *CsSWEET17*-RNAi calli to investigate its effects on sucrose content. The expression levels of *CsSWEET17* (D) were analyzed using RT-qPCR, and sucrose content was measured (E). Data are presented as mean values ± SE (*n* = 3). Different letters (a, b, c, d) indicate significant differences using Duncan's Significant Difference test at *P* < 0.05 after one-way ANOVA. (F) A schematic model illustrates the molecular mechanism by which CsMYB36 regulates CsSWEET17-mediated sugar transport and accumulation in citrus fruit.

### CsMYB36 enhances the sucrose content by increasing the transcript level of *CsSWEET17*

While CsMYB36 has been demonstrated to positively regulate the transcription of *CsSWEET17*, its potential role in sugar accumulation requires further elucidation. Through Agrobacterium-mediated transformation of citrus juice sacs, we demonstrated that the OE of *CsMYB36* significantly enhanced *CsSWEET17* expression and markedly increased sugar content in the juice sacs (Fig. 6A and B). This elevation in sugar content was positively correlated with the upregulation of both genes (Fig. 6C). In contrast, silencing of *CsMYB36* in juice sacs led to a notable downregulation of *CsSWEET17* expression (Fig. 6A and B) and a corresponding decrease in sucrose levels (Fig. 6C). Additionally, based on RT-qPCR analysis, we successfully generated stable transgenic citrus calli with *CsMYB36* OE and RNAi knockdown, as well as transgenic tomato fruits with ectopic *CsMYB36* OE (Fig. 6D and G). Two positive transgenic lines were identified for each group. The expression levels of *CsMYB36* and *CsSWEET17* were either increased or decreased accordingly (Fig. 6E). Sucrose content was significantly higher in the *CsMYB36*-OE samples compared to the control group, while RNAi knockdown lines exhibited the opposite phenotype (Fig. 6F). Similarly, the tomato fruit phenotype demonstrated that *CsMYB36* OE significantly elevated sucrose levels (Fig. 6H). We further analyzed the promoter of tomato *SlSWEET17*, which contains a binding site of MYB (Supplementary Fig. S6A). The Y1H experiment also demonstrated that CsMYB36 can bind to the *SlSWEET17* promoter (Supplementary Fig. S6B). The transcript level of *SlSWEET17* in *CsMYB36* overexpressing tomato was increased accordingly, implying the potential conserved regulation mechanism of MYB36-SWEET17 module between citrus and tomato (Supplementary Fig. S6C). The subcellular localization analysis of SlSWEET17 confirmed its predominant plasma membrane localization (Supplementary Fig. S7A). Functional complementation assays using CSY4000 presented the similar yeast growth phenotype, as the yeast expression with SlSWEET17 can growth well in the sucrose medium, but not in glucose and fructose mediums (Supplementary Fig. S7B). This observation demonstrated that SlSWEET17, akin to CsSWEET17 in citrus, selectively facilitated sucrose transport. These results provided compelling biological evidence supporting the positive regulatory role of CsMYB36 in promoting sucrose accumulation by upregulating *SWEET17* expression.

### Ca^2+^ and CsMYB36 induced the sugar accumulation depends on the *CsSWEET17*

To investigate the genetic relationship between CsMYB36 and CsSWEET17, the co-transformation of citrus calli was employed for gene expression and sucrose content assay. OE of *CsMYB36* in the control calli with an EV-Ri significantly increased sucrose content. Nevertheless, the sucrose accumulation effect was abolished when *CsMYB36* was overexpressed in the *CsSWEET17*-RNAi background (Fig. 7A–C). To further verify the role of CsSWEET17 in Ca^2+^-induced sucrose accumulation, the sucrose content was detected both in control (EV-Ri) and *CsSWEET17*-RNAi transgenic calli samples under Ca^2+^ treatment. The results indicated that Ca^2+^ induced higher *CsSWEET17* expression level and increased sucrose content in the control calli, but not in the *CsSWEET17*-RNAi lines (Figs. 7D and E), implying that the Ca^2+^-induced sucrose accumulation relied on the expression of *CsSWEET17*. Collectively, these findings provide strong biological and genetic evidence that Ca^2+^ and CsMYB36 regulate sucrose accumulation via a mechanism that is dependent on the *CsSWEET17*.

## Discussion

It is widely recognized that carbohydrate metabolism and transport are among the most critical factors determining the formation and maintenance of horticultural fruit quality [44]. Carbohydrates are primarily synthesized in the mesophyll cells of leaves and mainly transported to citrus fruit in the form of sucrose. Therefore, the strength of the fruit sink plays a crucial role in the accumulation of soluble sugars in the fruit, with the vacuole being the primary storage site in citrus. Most recent researches have focused on how the photosynthetic products transport into the fruit vacuole [41, 45, 46], while the unloading mechanism from the vascular bundles clustered in fruit peel remains largely unexplored. Gene expression analysis of Ca^2+^-treated citrus fruit and calli, and various transformation methods indicate that CsSWEET17, which was transcriptionally induced by CsMYB36 in response to Ca^2+^ treatment, plays an important role in sucrose transport from the citrus fruit peel tissue during early citrus fruit development. These insights enhance our understanding of sugar accumulation in citrus fruit and provide valuable information on the transcriptional regulation and transport function of CsSWEET17.

As a typical member of the SWEET transporter family, CsSWEET17 exhibits evolutionarily conserved sequence motifs and transmembrane topology characteristic (Supplementary Fig. S2). Functional characterization through CSY4000 yeast mutant strain and *Xenopus laevis* oocyte system demonstrated that CsSWEET17 functions as an SUT (Fig. 2B–F, Supplementary Fig. S3C and D). Quantitative analysis of ^14^C-sucrose uptake kinetics further established pH 5.5 as the optimal condition for its transport activity (Fig. 2C), corresponding to the physiologically relevant pH range (4.5–7.0) documented for plant sugar transporters [47, 48]. This pH-dependent transport mechanism is mediated by the electrochemical proton gradient across membranes, wherein the proton motive force facilitates co-transport of sucrose with hydrogen ions, consistent with established models of secondary active transport [49]. However, recent studies have demonstrated that *AtSWEET17* is expressed in the root parenchyma and vascular tissues of *Arabidopsis thaliana*, where it facilitates fructose transport under drought conditions to maintain cytosolic fructose homeostasis [14, 16]. SlSWEET17 localizes to the plasma membrane, small vacuoles and Golgi apparatus, yet it lacks the capacity to transport glucose or fructose [29]. Building upon previous findings, we conducted systematic subcellular localization studies of CsSWEET17. The results demonstrated its polytopic distribution across plasma membranes, tonoplasts small vesicles (Supplementary Fig. S3A). Subsequent functional validation through the W303 yeast system and esculin uptake assays demonstrated sucrose transport activity (Supplementary Fig. S3B), thereby confirming its operational capacity in vacuolar membrane. Although CsSWEET17 primarily facilitates sucrose transport, its functional role may adapt to environmental or metabolic demands. This functional plasticity is further exemplified by divergence among SWEET family members: AtSWEET16 transports sucrose in *Arabidopsis* [50], whereas grape VvSWEET15 exhibits dual specificity for both sucrose and fructose [28]. Notably, transported sucrose is rapidly hydrolyzed into hexoses by vacuolar acid invertase, establishing a sucrose concentration gradient between the cytoplasm and vacuole that facilitates further transport, a mechanism also observed in beet roots [51]. In addition, multi-system functional validation through transient expression in citrus juice sacs, homologous OE in calli, and heterologous expression model in tomato fruit consistently confirmed CsSWEET17's capacity to promote sucrose accumulation in storage compartments (Fig. 3A–F). The observed interspecies functional divergence among SWEET17 orthologs highlights evolutionary adaptations enabling lineage-specific optimization of carbohydrate partitioning strategies.

Gene expression regulation has profound effects throughout the plant lifecycle, with transcriptional regulation serving as a pivotal mechanism for controlling plant growth and development and all other physiological activities [52]. Nevertheless, the regulatory pathway governing sucrose metabolism in fruit sweetness development remains poorly resolved, with only a handful of transcription factors functionally validated. Elucidating the upstream regulatory network of sugar transport proteins can offer valuable theoretical insights into enhance the quality of citrus fruits. According to gene-expression association analysis and Y1H assay, CsMYB36 was identified to bind to and enhance the transcriptional activity of the *CsSWEET17* promoter in response to Ca^2+^ treatment. Furthermore, LUC, EMSA and ChIP-qPCR analyses demonstrated that CsMYB36 binds to the TTAGGT site on the *CsSWEET17* promoter and activates its expression (Fig. 5E–G). In addition, the transgenic results demonstrated that the OE of *CsMYB36* induced the higher sucrose content both in citrus and tomato fruits (Fig. 6). To confirm whether the increased sugar content in tomato fruit with the OE of *CsMYB36* is related to *SlSWEET17*, Y1H analysis verified that CsMYB36 can bind to the SlSWEET17 promoter (Supplementary Fig. S6B). Moreover, RT-qPCR analysis revealed that *SlSWEET17* expression is upregulated in *CsMYB36*-OE tomato fruits (Supplementary Fig. S6C), suggesting that the module interaction of MYB36*-SWEET17* and its function are conserved between the two species. Integrated analyses of transgenic citrus calli of *SWEET17*-Ri with transiently OE of *CsMYB36* or treated with Ca^2+^ revealed that the sucrose accumulation induced by Ca^2+^ or CsMYB36 depend on the transcript level of *CsSWEET17* (Fig. 7A–E). Emerging evidence indicates that transcription factors function as Ca^2+^ signal responders to coordinate physiological interactions. For example, in apple fruit, Ca^2+^/MdCDPK7 suppresses ethylene biosynthesis by modulating the activity of MdMADS5 and MdACO1, thus delaying fruit ripening [53]. We hypothesize that a similar regulatory mechanism involving Ca^2+^ sensors and protein kinases may govern the ‘*CsMYB36–CsSWEET17*’ module to control sucrose accumulation in fruit, which warrants further mechanistic investigation.

Sugars in fruits are derived from photosynthetic products assimilated by the leaves [1]. The juice sacs, which are the fastest growing tissues in the fruit, serve as the primary storage site for photosynthetic products [54]. The peel of citrus fruits has a unique structure: the outer layer of the peel is the oil layer, which contains chlorophyll and has photosynthetic ability, while the inner layer is the white flesh layer, which lacks photosynthetic capacity [40]. Existing studies suggest that the white flesh layer may be involved in unloading sugars. Before the fruit reaches full maturity, the oil layer retains its photosynthetic capability. Photosynthetic activity within the fruit peel exhibits a progressive decline during developmental progression, tightly coupled with chlorophyll catabolism and structural maturation. The photosynthetic products in the peel are primarily used for its own development, which not only reduces the peel’s dependency on assimilates from the leaves but also contributes to sugar accumulation in the juice sacs [55]. The vascular bundles terminate near the epidermis of the segments, but a layer of white flesh cells exists between the vascular bundles and the epidermis. Therefore, it is expected that the phloem unloading of sugars primarily occurs in the white flesh cells before the sugars reach the fruit pulp [54]. However, few studies have explored the key regulatory mechanisms underlying sugar unloading from the citrus peel into the juice sacs. Our RT-qPCR results show that both *CsSWEET17* and *CsMYB36* are specifically expressed in the peel, with relatively lower expression in the juice sacs and other tissues. Additionally, their expression levels decrease progressively during fruit development. This pattern mirrors the decline in photosynthetic activity in the peel during development, suggesting that the CsMYB36–CsSWEET17 module regulates phloem-mediated sucrose efflux during early fruit development, establishing its dominance in carbohydrate partitioning from the peel to the juice sacs sink.

Based on the observed regulatory pattern, we propose that during sweet orange fruit development, the ‘CsMYB36–*CsSWEET17*’ module facilitates sucrose unloading and transport from the peel to the juice sacs, with Ca^2+^-treatment augmenting the positive transcriptional regulation of *CsSWEET17* by CsMYB36 (Fig. 7F). This results in significant sucrose accumulation in citrus fruits, thereby promoting fruit development and ripening. In conclusion, these findings expand our understanding of the function and regulatory mechanisms of sugar transporters in citrus, offering valuable insights into the ‘CsMYB36*–CsSWEET17*’ module’s role in regulating sugar transport for improving fruit quality.

## Materials and Methods

### Plant materials and growth conditions

Navel orange (*C. sinensis* Osb. var. *brasliliensis* Tanaka) fruits were cultivated and managed in the National Center of Citrus Breeding, Huazhong Agricultural University, Wuhan, China. The fruits were used for Ca^2+^ treatment, RNA isolation, gene cloning and expression analysis. ‘Guoqing NO.1’ citrus calli and ‘MicroTom’ tomato (*Solanum lycopersicum* cv. Micro Tom) were used for gene transformation. The juice sacs of HB pummelo (*C. maxima*) were employed for transient expression assays of various genes. Citrus calli was cultured on solid Murashige and Tucker (MT) medium supplemented with 40 g/L sucrose and 8 g/L agar, incubated at 25°C in the dark. The ‘MicroTom’ tomato plants and tobacco plants (*Nicotiana benthamiana*) were grown in a growth chamber. The growth chamber was maintained at 24°C, 65% relative humidity, with a photoperiod of 16 h light and 8 h dark.

### Ca^2+^ treatments and Ca^2+^ content determination in citrus fruits and calli

In this study, three selected plants of Navel orange (*C. sinensis* Osb. var. *brasliliensis* Tanaka) had similar vigor, were free of pests and diseases, and were managed uniformly. The Ca^2+^ treatment protocol involved periodic fruit soaking treatments using an 81 mM aqueous solution of Ca^2+^ nitrate tetrahydrate [Ca(NO_3_)_2_·4H_2_O]. Applications were initiated at 60 days after full bloom (DAFB) and continued at 10-day intervals until two weeks prior to harvest, with treatments deliberately scheduled to avoid rainy days. The distilled water treatment served as a control (CK). Three fruits per tree were randomly collected from four directions at the same height of the canopy to ensure that the fruits were similar in size, undamaged, and free of obvious pest and disease symptoms. Sampled fruits were analyzed for sugar content and gene expression, and three biological replicates were set up for physiological measurements. Healthy and uncontaminated calli were selected as experimental materials. The Ca^2+^ treatment involved culturing the calli in MT medium supplemented with 3 mM Ca(NO_3_)_2_. The culturing calli on standard MT medium works as a CK. Each treatment consisted of three biological replicates, with sampling conducted two weeks after treatment. After sampling, the tissues were rapidly frozen in liquid nitrogen for gene expression and sugar content analysis. Each sample included three biological replicates.

Ca^2+^ content was analyzed following a previously established protocol [56]. Briefly, approximately 100 mg of dried plant material was digested with an HNO_3_–HClO_4_ acid mixture (4:1, v/v) under low-temperature conditions until complete clarification. The digestate was diluted to 50 ml with deionized water, and Ca^2+^ concentrations were quantified via inductively coupled plasma optical emission spectrometry (ICP-OES; Agilent 5110VDV, USA) using matrix-matched calibration standards.

### Analysis of sugars content via gas chromatography method

The gas chromatography (GC) method for analyzing sugars was conducted following previously published protocols [41]. Samples were ground into a powder in liquid nitrogen prior to extraction. Each sample (0.2 g) was extracted using 1.4 ml of 75% (v/v) methanol and chloroform. Ribitol (0.12 mg) was added as an internal standard during the extraction process. Following extraction, samples were sequentially derivatized with methoxyamine hydrochloride and N-methyl-trimethylsilyl-trifluoroacetamide. The analysis was performed using the GC 9720plus Gas Chromatography Workstation (FULI INSTRUMENTS, Hangzhou, China), equipped with a flame ionization detector and a non-polar RBX-5 (5%-phenyl)-methylpolysiloxane column (30.0 m × 0.32 mm × 0.25 μm), with quantification carried out using the internal standard method (FL97Plus.lnk). Each sample was analyzed in triplicate.

### Phylogenetic tree construction and multiple sequence alignments

The amino acid sequences of SWEET members previously reported in Arabidopsis were downloaded from the TAIR database (https://www.arabidopsis.org/), while sequences of citrus SWEET were obtained from CPBD (http://citrus.hzau.edu.cn/). The resulting sequences were input into MEGA X64 software for phylogenetic tree construction and visualized using the iTOL website (https://itol.embl.de/). Multiple sequence alignments were conducted using MEGA, and visualizations were performed with GeneDoc 2.7 (https://genedoc.software.informer.com/2.7/).

### RNA extraction, cDNA synthesis and RT-qPCR analysis

Total RNA was extracted from citrus fruits, calli or tomato fruit samples using the EASYspin Plus RNA Extraction Kit (Aidlab Biotech, Beijing, China) according to the manufacturer’s instructions. Genomic DNA was removed and complementary DNA (cDNA) was synthesized using the EasyScript One-Step gDNA Removal and cDNA Synthesis SuperMix (Transgen Biotech, Beijing, China). RT-qPCR was conducted using SYBR qPCR Master Mix (Vazyme Biotech, Nanjing, China) on a Real-Time PCR system (Applied Biosystems, Foster City, CA, USA). The total reaction volume was 10 μl, which included 5 μl of 2× SYBR Green qPCR Master Mix, 0.3 μl of forward and reverse primers (each at 10 μM), and 200 ng of template cDNA. The relative gene expression levels were calculated using the 2^-ΔΔCt^ method [57]. Each result was obtained from three biological replicates. T2 fruits from wild-type (WT) and overexpressing tomato lines were sampled for RT-PCR analysis. The *SlActin* gene from tomato was used as an internal control. The PCR annealing temperature was set at 60°C, with 27 cycles and an extension time of 5 s. Primers used for RT-qPCR or RT-PCR are listed in Supplementary Table S1.

### Subcellular localization assay

Using cDNA from citrus fruit as a template, the full-length coding sequences (CDS) of *CsSWEET17* (705 bp) and *CsMYB36* (1011 bp) excluding the stop codon were amplified via PCR, and then cloned into the vectors pRI101-GFP and pEYFP101, respectively. The constructed vectors and the EV were transformed into *A. tumefaciens* GV3101. Vectors containing the mCherry-labeled CBL1n-ORF [58] and mCherry-labeled VirD2NLS [59] were co-transformed as markers for plasma membrane localization and nucleus localization, respectively. A needle-free syringe was used to inject the suspension (0.01 M MES, 0.01 M MgCl_2_, 50 μM acetosyringone, and OD_600_ = 0.6–0.8) into the abaxial surface of tobacco leaves. After 72 h, the fluorescent signals were observed using a confocal microscope (Leica TCS-SP8, Mannheim, Germany). The GFP signal was excited at 488 nm and detected using a bandpass filter from 500 to 540 nm. The excitation wavelength of the mCherry signal is 552 nm and the emission wavelength is from 599 to 646 nm. Chlorophyll autofluorescence was detected at 590–650 nm emission under 552 nm excitation. The primers used in this study are listed in Supplementary Table S1.

### Functional characterization of sugar transport through heterologous expression in yeast

To perform complementation assays in yeast (*Saccharomyces cerevisiae*), the CDS of *CsSWEET17* was amplified by PCR using cDNA from citrus fruit as a template. The amplified fragment was then inserted into the pDR196 vector, which had been linearized with *EcoR*I/*Sal*I. The recombinant vector and the EV were transformed into the hexose-transport-and SUC2-deficient yeast strain CSY4000 [60, 61]. The transformed yeast cell suspension was diluted in a gradient and cultured on solid SC/-Uracil medium containing either 2% (w/v) maltose (control) or 2% (w/v) sucrose/glucose/fructose, at 30°C for 3 days. Sugar uptake assays were conducted as described in previous reports [62].

Esculin fluorescence-based sugar transport assay. To investigate sucrose transport capability, W303 yeast cells were co-transfected with CsSWEET17-pDR196 and the SUT construct StSUT1-pDR196. An optimized protocol derived from [41] was employed for esculin (a sucrose analog) uptake analysis. Transformants were grown in SD-Ura medium supplemented with 2% glucose (w/v) to mid-log phase (OD_600_ = 0.6–0.8). Following PBS (pH 5.0) washing and resuspension (OD_600_ = 0.5), cells were exposed to 1 mM esculin at 30°C for 2 h. Subcellular esculin distribution and fluorescence intensity were monitored via confocal microscopy (Leica TCS SP8; excitation 405 nm, emission 440 nm).

### ^14^C-sugar uptake assay in yeast

Yeast strains carrying the recombinant plasmid or pDR196 were grown in liquid YPA medium supplemented with 2% (w/v) maltose to an OD_600_ value of 0.8, then harvested by centrifugation at 5000 rpm and washed twice with 25 mM phosphate-buffered saline (PBS, pH 5.5). The collected yeast cells were resuspended in the same PBS buffer to an OD_600_ value of 20 (0.1 mg yeast/100 μl) for the sugar uptake assay. To detect sugar uptake, ^14^C-Suc (0.02 μCi) was added to the yeast cells with the final concentration of 100 mM unlabeled sucrose substrate, and the mixture was incubated in a shaking water bath at 30°C. After the incubation period, the cells were washed three times with 1 ml of cold distilled water. The filters were placed into scintillation (Ecoscint H) and measured using a multifunctional scintillation counter (Tri-Carb 2810 TR, PerkinElmer, USA).

### Quantitative ^14^C-sugar uptake profiling in *Xenopus laevis* oocytes

The coding sequence of CsSWEET17 was amplified by PCR and cloned into *Xenopus laevis* oocyte expression vectors using a uracil excision-based cloning technique [62]. cRNA was synthesized using the mMessage mMachine T7 transcription kit (Thermo Fisher, USA), and 50 nl of cRNA was microinjected into each oocyte. Injected oocytes were cultured in Ca^2+^-free ND96 solution (96 mM NaCl, 2 mM MgCl₂, 1 mM KCl, 5 mM HEPES (pH 7.5 adjusted with NaOH), 0.1 mg ml^−1^ gentamycin and 0.1 mg ml^−1^ streptomycin) for 48-h protein expression. Water-injected oocytes served as negative controls. For the sugar uptake assay, a modified protocol from [63] was employed. At 40 h post-cRNA injection, oocytes were incubated with ^14^C-sucrose (0.02 μCi) and 100 mM unlabeled sucrose in ND96 buffer at 30°C with gentle shaking. Following incubation, oocytes were washed three times with 1 ml of ice-cold distilled water, lysed in 1% SDS and transferred to scintillation fluid (Ecoscint H). Radioactivity was quantified using a Tri-Carb 2810 TR multipurpose scintillation counter (PerkinElmer, USA). Three independent biological replicates were performed, yielding consistent results. Primer sequences are provided in Supplementary Table S1.

### Genetic transformation of citrus calli

The CDS of *CsSWEET17* and *CsMYB36* (without stop codons) were amplified using citrus fruit cDNA as template. The fragments were then purified and cloned into the pENTR-1A-flag vector using the ClonExpress II One-Step Cloning Kit (Vazyme, Nanjing, China) at *Sal*I*/BamH*I restriction sites. Subsequently, the full-length CDS was inserted into the pK7WG2D vector using LR Clonase™ enzyme (Thermo Fisher, Waltham, MA, USA) via a Gateway reaction for gene OE (OE). For the construction of the RNAi vector, the amplified CDS fragments were first inserted into the pDONOR221 vector using BP Clonase™ enzyme (Thermo Fisher, Waltham, MA, USA) via a Gateway reaction. The sequences were then transferred to the pK7GWIWG2D vector using LR Clonase™ enzyme (Thermo Fisher, Waltham, MA, USA). The OE (OE) constructs, RNAi constructs, and EVs were introduced into *A. tumefaciens* strain EHA105 (Weidibio, Shanghai, China) for calli transformation. After removing the excess liquid from the suspended calli cultures using filter paper, the calli was placed in MT liquid medium supplemented with 50 μM acetosyringone containing different plasmid constructs from the EHA105 strain. The culture was mixed with the calli at an optical density (OD_600_) of 0.6 and subjected to vacuum infiltration for 15 min, followed by shaking at 28°C for 10 min. The infected citrus calli was co-cultivated with Agrobacterium for 3 days and then washed three times with sterile water. The collected calli samples were placed in MT medium supplemented with 40 g/L sucrose, 250 mg/L carbenicillin and 50 mg/L kanamycin and cultured for 4–8 weeks until positive calli emerged. The positive calli was then subcultured in MT medium for further experiments. The primers used are listed in Supplementary Table S1.

### Genetic transformation of tomato plant

The transformation of tomato plants was conducted using previously published methods [41]. The pK7WG2D-*CsSWEET17* and pK7WG2D-*CsMYB36* vectors were introduced into *A. tumefaciens* strain GV3101 (Weidibio, Shanghai, China) for the purpose of heterologous OE in tomato cotyledons. After sterilization, the tomato seeds were germinated on 1/2 MS medium supplemented with 10 g/L sucrose for 6–7 days. The enlarged cotyledons were then pre-cultured on MS medium for 2 days, with both ends trimmed. The cotyledons were incubated with the Agrobacterium-mediated pK7WG2D gene vectors at 28°C for 10 min. The infected explants were selected on kanamycin-resistant MS medium containing 2 mg/L trans-zeatin, 0.1 mg/L IAA and 200 mg/L Timentin to obtain positive regenerants for further experiments. The regenerated tomato plants were subsequently transplanted into soil, and seeds were collected from these plants. The second generation (T2) fruits derived from transgenic plants were used for subsequent experiments.

### Transient infiltration of citrus juice sacs

The recombinant pK7WG2D vector for OE is the same as the recombinant pK7WG2D vector for calli transformation. VIGS was performed based on the TRV-RNA1 (pTRV1) and TRV-RNA2 (pTRV2) vectors [64]. The CDS fragment of target gene was inserted into the TRV2 vector, which was linearized using *BamH*I/*Sma*I sites, thereby constructing the VIGS vector. The OE, VIGS constructs and EV were introduced into Agrobacterium EHA105 (Weidibio, Shanghai, China) for transient infiltration. Agrobacterium was cultured at 28°C until an OD_600_ of 0.8 reached. The bacterial culture was harvested by centrifugation at 6000 rpm and resuspended in an infiltration buffer containing 0.01 M MES, 0.01 M MgCl_2_ and 50 μM acetosyringone. For VIGS, the pTRV1 and pTRV2 constructs were mixed in a 1:1 ratio, with pTRV1-EV and pTRV2-EV serving as controls. The resuspended culture was mixed with the juice sacs and subjected to 30 min of vacuum infiltration. The infected juice sacs were then co-cultivated with Agrobacterium in MS medium supplemented with 30 g/L sucrose, 5 mg/L vitamin C and 50 μM acetosyringone for 4 days. The primers used are listed in Supplementary Table S1.

### LUC assay

The CDS of *CsMYB36* was cloned into the pGreenII 0800 vector pGreenII 62-SK vector, which was linearized using the *BamH*I/*Kpn*I restriction sites. Additionally, the promoter sequence of CsSWEET17 was cloned into the pGreenII 0800 vector, also linearized with *BamH*I/*Kpn*I, to drive the expression of LUC. The recombinant vectors were transformed into Agrobacterium GV3101 containing p19. For the experimental setup, Agrobacterium solutions containing CsSWEET17pro-0800/EV-SK and CsSWEET17pro-0800/MYB35-SK were injected into 4-week-old tobacco leaves. After 2–3 days, the substrate D-fluorescein potassium salt was applied, and fluorescence intensity was detected using an in vivo imaging system (NightSHADE L985, Berthold Technologies, Germany). The LUC and renilla (REN) enzyme activities in the injected tobacco leaf samples were quantified using an LUC reporter gene detection kit (Yeasen, Shanghai, China). The primers used are listed in Supplementary Table S1.

### Y1H assay

Different-length fragments of *CsSWEET17* promoter (CsSWEET17pro) generated by PCR were cloned into the pAbAi vector using primers listed in the Supplementary Table S1. The CDS of Cs*MYB36* was cloned into the pGADT7 vector to create the CsMYB36-pGADT7 construct, which was then transformed into the yeast strain Y1H Gold containing the CsSWEET17pro-pAbAi construct. The co-transformed positive clones were spotted on a series of dilution factors (1:1, 1:10, 1:100 and 1:1000) onto SD-Ura-Leu plates, both with and without varying concentrations of AbA, to suppress promoter auto-activation. The plates were incubated at 30°C for 2 to 3 days. The p53-pGADT7 and p53-pAbAi constructs served as positive control, while EV-pGADT7 and CsSWEET17pro-pAbAi served as negative control.

### ChIP-qPCR assay

Citrus calli transformed with the pK7WG2D-flag vector served as the control group, while the pK7WG2D-CsMYB36-flag vector was used as the test group. Samples were mixed with 1% (w/v) formaldehyde solution and subjected to vacuum fixation. After grinding in liquid nitrogen, nuclei were extracted via filtration and sucrose density gradient centrifugation. Protein-DNA complexes were immunoprecipitated using anti-flag Magnetic Beads (GenScript, L00835, Nanjing, China). Following reverse crosslinking, DNA was extracted for qPCR analysis. Primers used for qPCR are listed in Supplementary Table S1.

### Recombinant protein purification and EMSA

The full-length CDS of *CsMYB36* was cloned into the pGEX-4 T-1 vector and transformed into the *Escherichia coli* strain Rosetta (DE3) (Weidibio, Shanghai, China) for protein expression. Transformed cells were cultured in LB medium until the OD_600_ reached 0.6. Induction was then carried out at 16°C for 20 h in the presence of 0.5 mM isopropyl β-D-1-thiogalactopyranoside (IPTG). Cells were collected by centrifugation and resuspended in buffer (20 mM Tris–HCl, pH 8.0, 0.5 M NaCl). The suspension was subjected to ultrasonic treatment on ice using a 3 s on/2 s off cycle for 15 min, followed by centrifugation at 10 000 rpm for 20 min at 4°C to collect the supernatant, where the fusion protein was induced. Probes containing MYB binding motifs were synthesized and biotinylated (Sangon Biotech, Shanghai, China). Unlabeled probes were used as competitors. All probes were annealed before used in EMSA. The probes were incubated with the fusion protein in 10 μl of reaction mixture for 30 min. The reaction mixture was then electrophoresed on a 6% native polyacrylamide gel and transferred to a nylon membrane (Biosharp, Hefei, China). After UV cross-linking, the membranes were subjected to blocking, HRP ligation, washing and equilibration steps using a chemiluminescent EMSA kit (Beyotime Biotech, GS009, Shanghai, China). Finally, the migration of biotinylated probes on the membrane was observed using a chemiluminescence imaging system (Tanon 5200, Shanghai, China). The primers used are listed in Supplementary Table S1.

### Statistical analysis

Statistical analysis was performed using SPSS version 26.0.0.2. Data are presented as means ± standard deviation from three biological replicates. Asterisks indicate statistically significant differences determined by Student’s t-test (^*^0.01 < *P* < 0.05; ^**^P < 0.01; ^***^P < 0.001). Different letters (a, b, c) denote significant differences as assessed by one-way analysis of variance (ANOVA).

### Accession numbers

For genes in citrus (http://citrus.hzau.edu.cn/, Citrus sinensis v3.0): *CsSWEET17* (Cs_ont_9g002700), *CsMYB36* (Cs_ont_8g005880), *CsERD6L5* (Cs_ont_3g017200), *CsSWEET7* (Cs_ont_5g049860), *CsERD6L6* (Cs_ont_3g003580), *CsSTP10* (Cs_ont_7g018960), *CsSTP14* (Cs_ont_4g008780), *CsSTP13* (Cs_ont_9g005250), *CsbHLH123* (Cs_ont_7g004080), *CsMYB58* (Cs_ont_9g027950), *CsSPT* (Cs_ont_3g005320), *CsMYB62* (Cs_ont_5g036560), *CsNAC78* (Cs_ont_5g005590). For tomato gene (https://www.solgenomics.net/, Tomato ITAG release 4.0): *SlSWEET17* (Solyc01g099870), *SlMYB36* (Solyc06g074910), *SlACTIN* (Solyc03g115810).

## Supplementary Material

Web_Material_uhaf175

## Data Availability

All data is available within the manuscript and its supporting materials.
