# Supplementary material for: The CsMYB36-CsSWEET17 module mediates the calcium-induced sucrose accumulation in citrus
Source: Hortic Res. 2025 Jul 16;12(10):uhaf175. doi: 10.1093/hr/uhaf175 (PMC12528650; doi:10.1093/hr/uhaf175)
Supplement: Web_Material_uhaf175 [file web_material_uhaf175.zip › 20250604-CsSWEET17 Supplementary figures.docx]

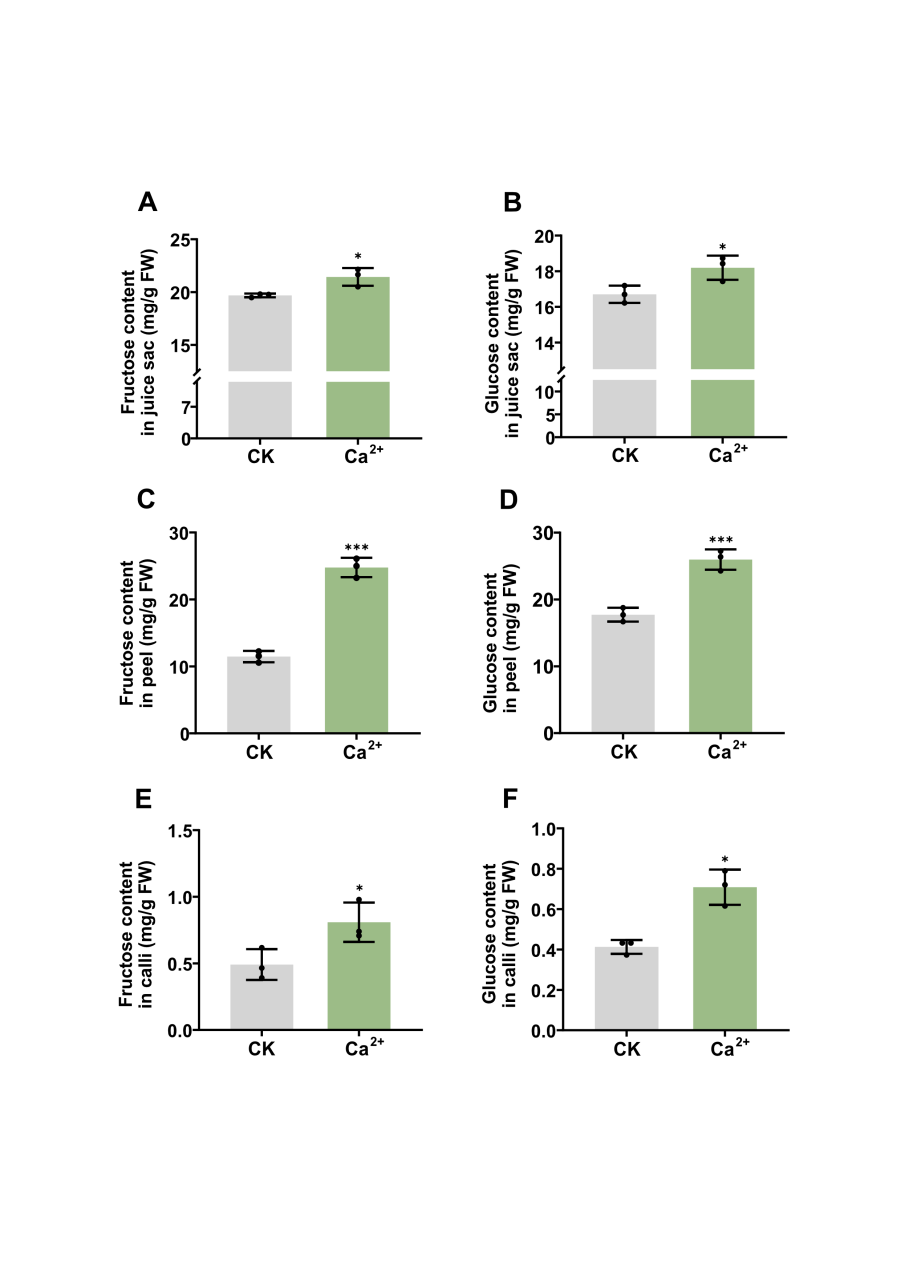


**Supplementary Figure S1. The content of fructose and glucose in calcium treated samples**

1. B) The fructose (A) and glucose (B) content in calcium treated juice sacs. (C-D) The fructose (C) and glucose (D) content in calcium treated peel of citrus fruit. (E-F) The fructose (E) and glucose (F) content in calcium treated citrus calli. Statistical analyses were performed on three biological replicates, with error bars representing standard error (SE) of the mean. Significant differences relative to control conditions are denoted by asterisks (*P < 0.05, ***P < 0.001; Student's t-test). CK represents the control.


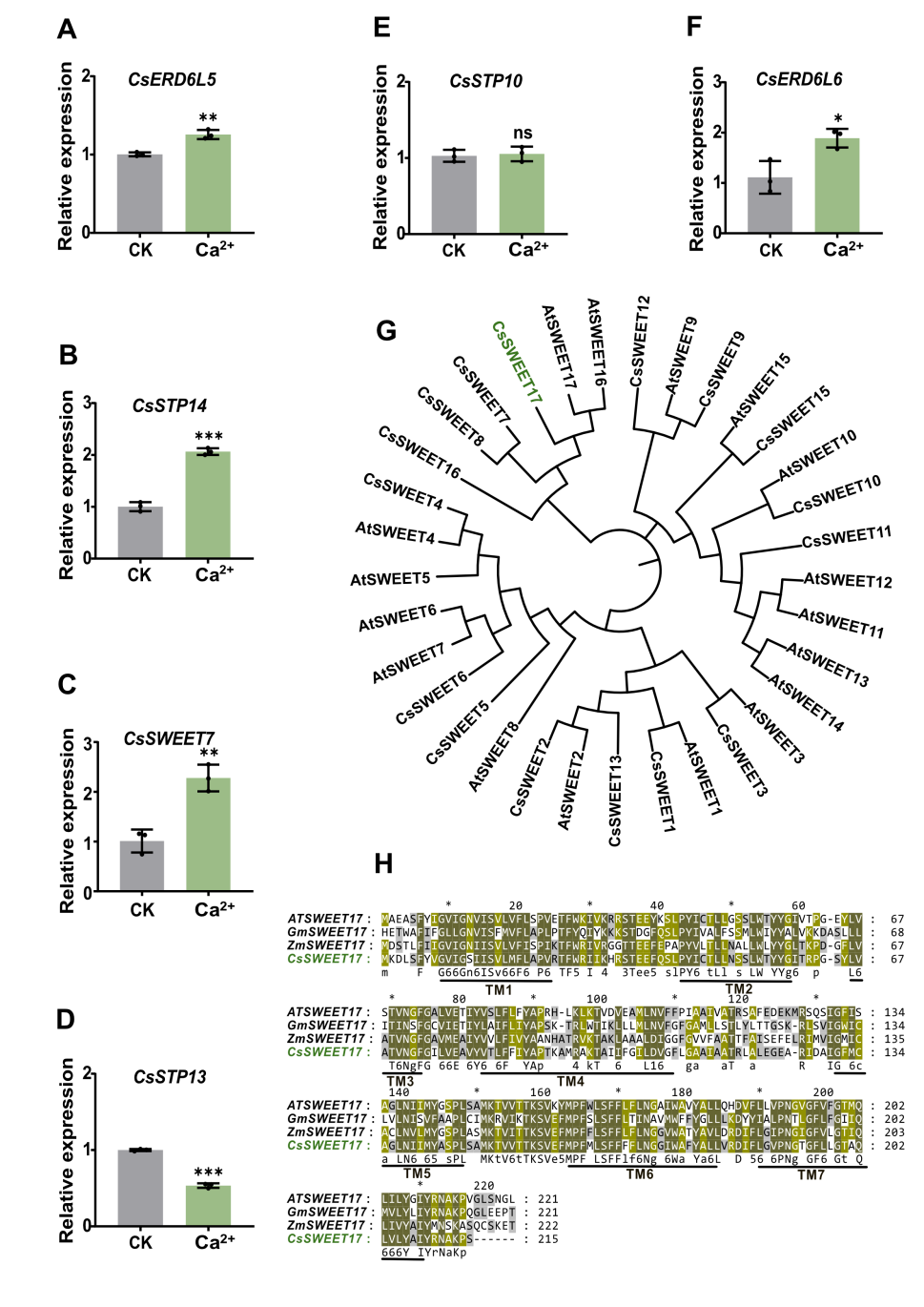


**Supplementary Figure S2. Selection and sequence analysis of *CsSWEET17***

(A-F) RT-qPCR validation of gene expression in calcium-treated calli. Error bars represent the standard error (SE) based on three biological replicates. Each data point corresponds to an independent biological repetition. Significant differences compared to the control are indicated by asterisks (*P < 0.05, **P < 0.01, ***P < 0.001, Student's t-test). (G) Phylogenetic relationships of SWEET proteins in sweet orange (*Citrus sinensis*) and *Arabidopsis thaliana*. A phylogenetic tree was constructed using the Maximum Likelihood method with amino acid sequence alignments performed in MEGA. (H) Multiple amino acid sequence comparison of CsSWEET17 with homologous proteins from *Arabidopsis thaliana* (AtSWEET17), *Glycine max* (GmSWEET17), and *Zea mays* (ZmSWEET17). Sequence alignment revealed conserved regions, with green shading indicating identical residues and light gray shading indicating conservative substitutions. These regions highlight the evolutionary conservation of the SWEET protein family across plant species.

_
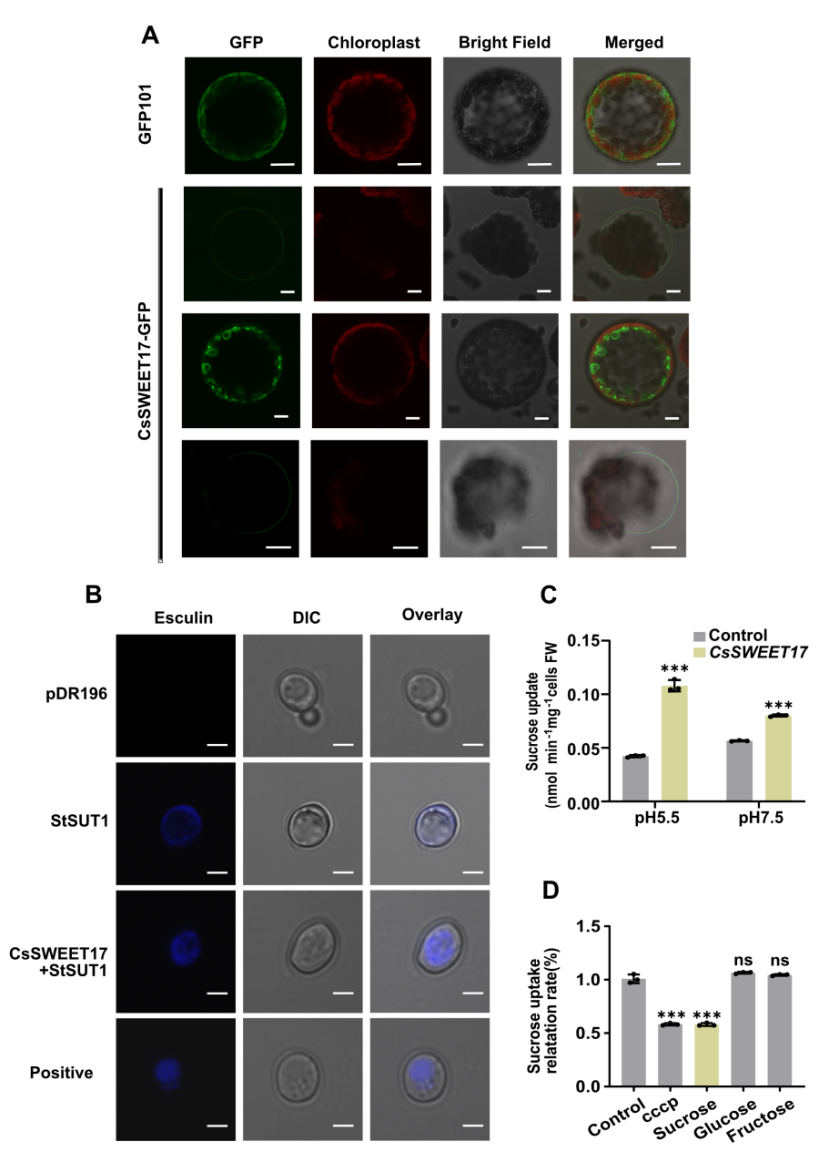
_

**Supplementary Figure S3. Subcellular localization of CsSWEET17 in protoplasts of *Nicotiana benthamiana* leaves and functional validation of sucrose transport activity**

1. The CsSWEET17 protein is localized to cell membrane, vacuolar membrane, and small vesicles. Confocal microscopy images of tobacco isolated protoplast and vacuole labeled with GFP fluorescent protein for CsSWEET17. Scale bars = 10 μm. (B) An esculin uptake assay was performed using yeast W303 cells. Under confocal microscopy, esculin exhibited cyan fluorescence. Expression of StSUT1, encoding a plasma membrane-localized sucrose transporter, resulted in esculin internalization into the cytosol. Following co-expression of StSUT1 and CsSWEET17, the cyan fluorescence signal was primarily localized within vacuoles. Postive control, CsTST2+StSUT1. No signal was detected in the empty vector control cells. Scale bars = 2 μm. (C) Investigation of pH-dependent sucrose uptake by CsSWEET17 in *Xenopus laevis* oocytes, with pDR196 (empty vector) used as a negative control. (D) ^14^C-Suc uptake of CsSWEET17 expressed in *Xenopus laevis* oocytes was studied in the presence of competing sugars.


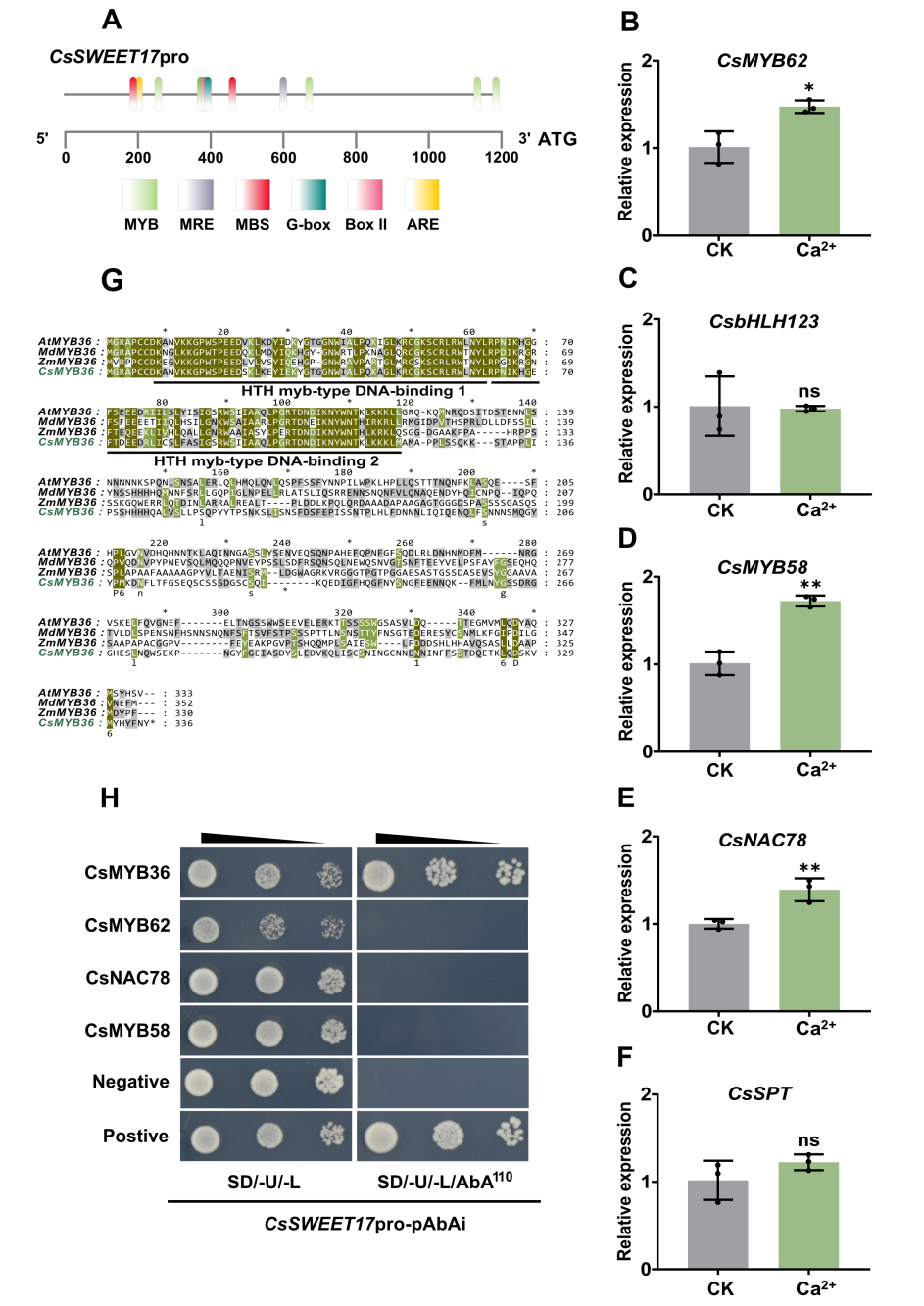


**Supplementary Figure S4. Selection and sequence analysis of *CsMYB36***

1. Predictive analysis of *cis*-acting regulatory elements within the promoter region of *CsSWEET17*. (B-F) RT-qPCR validation of transcription factor expression in calcium-treated calli. Error bars represent the standard error (SE) based on three biological replicates. Each data point corresponds to an independent biological repetition. Significant differences compared to the control are indicated by asterisks (*P < 0.05, **P < 0.01, ***P < 0.001, Student's t-test). (G) Multiple amino acid sequence comparison of CsMYB36 with homologous proteins from *Arabidopsis thaliana* (AtMYB36), *Malus domestica* (MdMYB36), and *Zea mays* (ZmMYB36). Sequence alignment reveals identical and conserved residues, with green shading indicating identical amino acids and light gray shading indicating conservative substitutions. (H) Y1H experiment demonstrating the interaction between *CsSWEET17* promoter and transcription factors. Positive control: pGBKT7-P53 and pGADT7-53. Negative control: SWEET17 promoter (pAbAi) and pGADT7 empty vector.


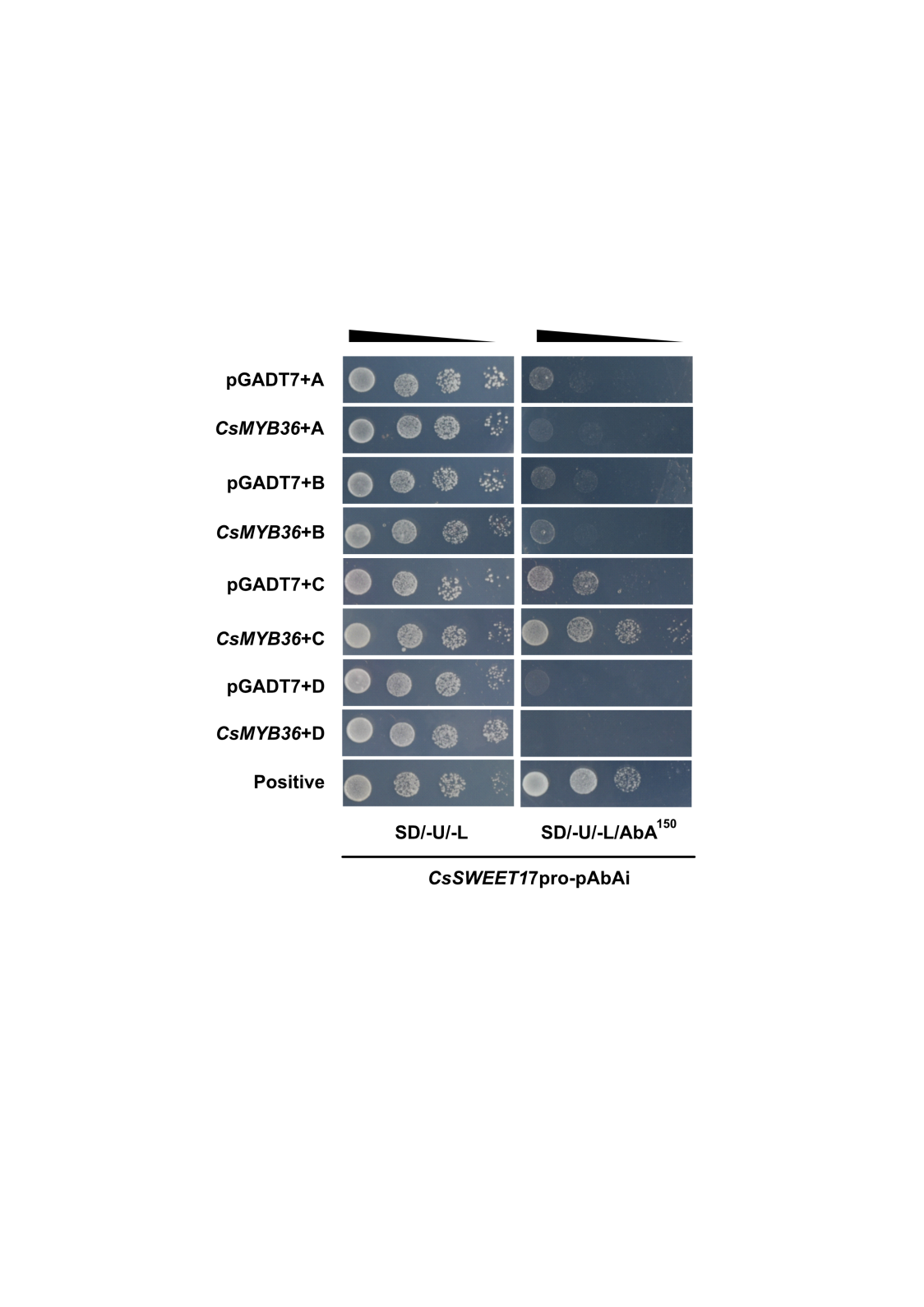


**Supplementary Figure S5.** **Y1H assay between CsMYB36 and different promoter segments of *CsSWEET17*.**

SD/-Ura/-Leu, SD medium without Ura and Leu; SD/-Ura/-Leu/AbA^150^, SD medium without Ura and Leu supplemented with AbA at the concentration of 150 mg/L. Positive control: pGBKT7-P53+pGADT7-53. The A-D denotes specific promoter regions of *CsSWEET17* tested in the Y1H experiment (Figure 3D).


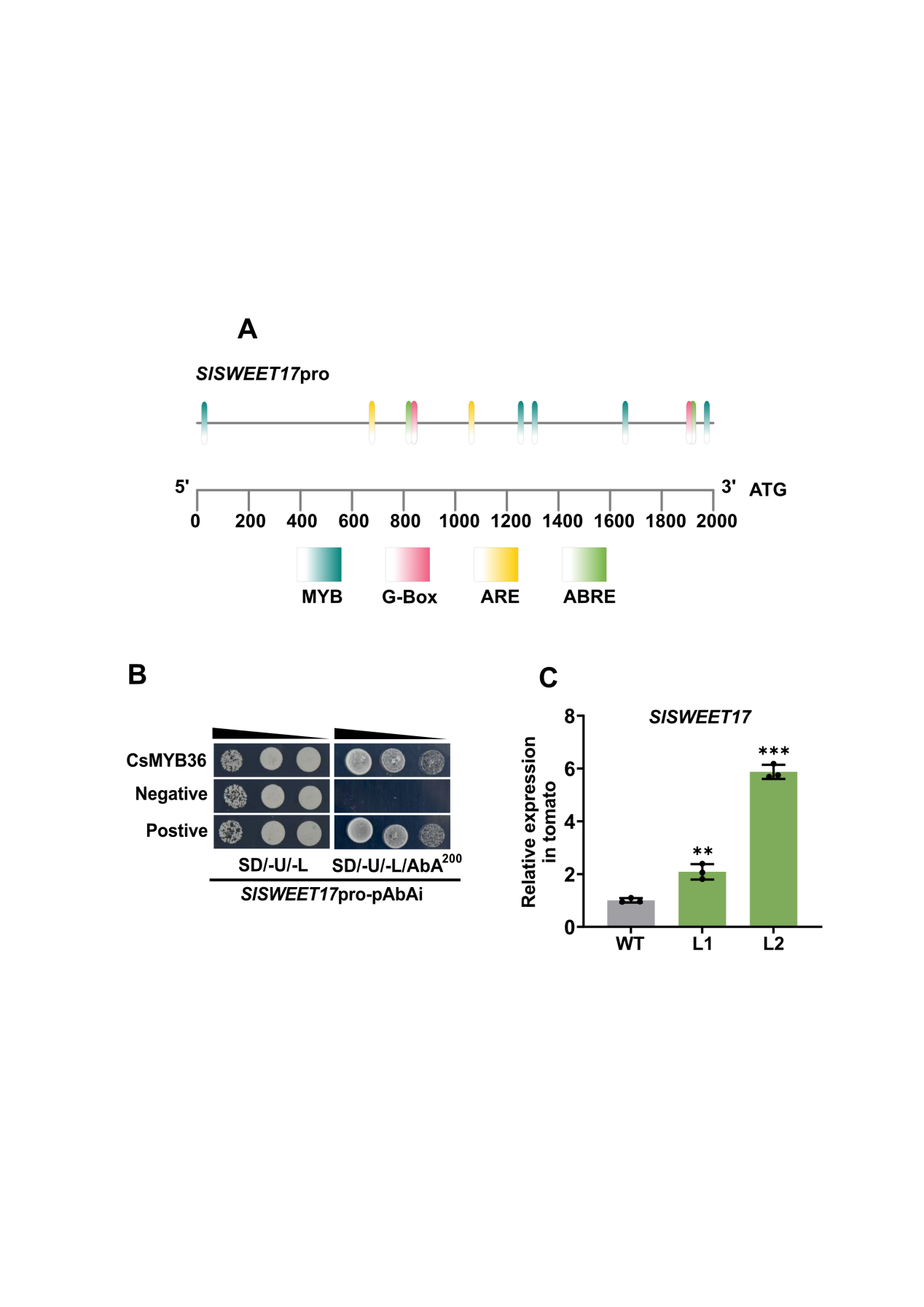


**Supplementary Figure S6. The relationship verification of CsMYB36 and *SlSWEET17* in tomato**

1. Predictive analysis of *cis*-acting regulatory elements in the promoter region of *SlSWEET17* (*SlSWEET17pro*). (B) Y1H assay showing the binding of CsMYB36 to the *SlSWEET17* promoter. SD/-Ura/-Leu, SD medium without Ura and Leu; SD/-Ura/-Leu/AbA^200^, SD medium without Ura and Leu supplemented with AbA at the concentration of 200 mg/L. Positive control: pGBKT7-P53+pGADT7-53; Negative control: *SlSWEET17*pro-pAbAi + pGADT7 empty vector. (C) RT-qPCR analysis of *SlSWEET17* expression in CsMYB36-OE transgenic tomato fruits. *SlActin* works as internal reference. L-1 and L-2, CsMYB36-OE tomato lines; WT, Wild Type, refers to the control. All the above results, each point represents an independent biological repeat and error bars means ± SE (n = 3). FW, Fresh weight. Asterisks indicate statistically significant differences compared with controls (one-way ANOVA with Tukey’s test, *P < 0.05, **P < 0.01, ***P < 0.001).


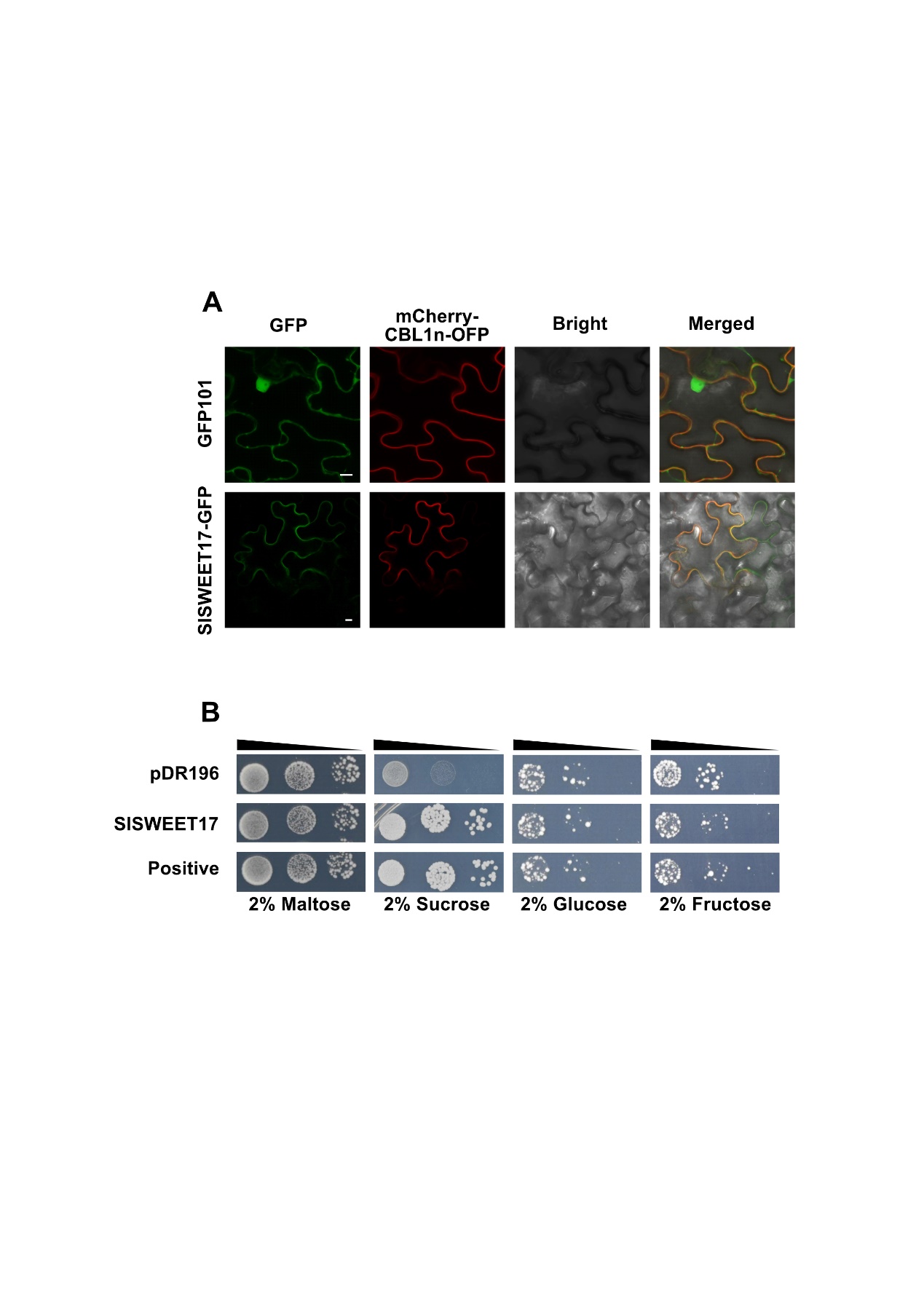


**Supplementary Figure S7. Validation of the plasma membrane localization of SlSWEET17 and its sucrose transport activity**

1. Fluorescence imaging of SlSWEET17 in transiently transformed *Nicotiana benthamiana* leaves demonstrated plasma membrane localization, supported by co-localization with a plasma membrane marker protein (mCherry-CBL1). Scale bars = 10 μm.

(B) SlSWEET17 restores sucrose uptake capability in the transporter-deficient yeast mutant CSY4000, as demonstrated by its ability to grow on selective sucrose medium, unlike the negative control carrying the empty pDR196 vector. Positive control, StSUT1-pDR196. The yeast growth was assessed after a 3-day incubation period.

**Supplementary Table S1. List of primers used in this study.**
